# Serum microRNA expression patterns that predict early treatment failure in prostate cancer patients

**DOI:** 10.18632/oncotarget.1776

**Published:** 2014-02-13

**Authors:** Prashant K. Singh, Leah Preus, Qiang Hu, Li Yan, Mark D. Long, Carl D. Morrison, Mary Nesline, Candace S. Johnson, Shahriar Koochekpour, Manish Kohli, Song Liu, Donald L. Trump, Lara E Sucheston-Campbell, Moray J. Campbell

**Affiliations:** ^1^ Department of Pharmacology & Therapeutics, Roswell Park Cancer Institute, Buffalo, NY; ^2^ Department of Cancer Prevention & Control, Roswell Park Cancer Institute, Buffalo, NY; ^3^ Department of Biostatistics & Bioinformatics, Roswell Park Cancer Institute, Buffalo, NY; ^4^ Department of Pathology, Roswell Park Cancer Institute, Buffalo, NY; ^5^ Department of Cancer Genetics, Roswell Park Cancer Institute, Buffalo, NY; ^6^ Department of Medical Oncology, Mayo Clinic, Rochester, MN; ^7^ Department of Medicine, Roswell Park Cancer Institute, Buffalo, NY

**Keywords:** Prostate cancer, microRNA, biochemical progression, miR-103, miR-125b, miR-222, cancer epigenetics

## Abstract

We aimed to identify microRNA (miRNA) expression patterns in the serum of prostate cancer (CaP) patients that predict the risk of early treatment failure following radical prostatectomy (RP). Microarray and Q-RT-PCR analyses identified 43 miRNAs as differentiating disease stages within 14 prostate cell lines and reflectedpublically available patient data. 34 of these miRNA were detectable in the serum of CaP patients. Association with time to biochemical progression was examined in a cohort of CaP patients following RP. A greater than two-fold increase in hazard of biochemical progression associated with altered expression of miR-103, miR-125b and miR-222 (*p* <.0008) in the serum of CaP patients. Prediction models based on penalized regression analyses showed that the levels of the miRNAs and PSA together were better at detecting false positives than models without miRNAs, for similar level of sensitivity. Analyses of publically available data revealed significant and reciprocal relationships between changes in CpG methylation and miRNA expression patterns suggesting a role for CpG methylation to regulate miRNA. Exploratory validation supported roles for miR-222 and miR-125b to predict progression risk in CaP. The current study established that expression patterns of serum-detectable miRNAs taken at the time of RP are prognostic for men who are at risk of experiencing subsequent early biochemical progression. These non-invasive approaches could be used to augment treatment decisions.

## 1 INTRODUCTION

In men in the USA and elsewhere, prostate cancer (CaP) is the most common noncutaneous cancer diagnosed and second leading cause of death [1, 2]. At multiple stages of disease there is ambiguity over progression risk and treatment responses. For example, the prostate-specific antigen (PSA) [3, 4] test has significantly increased cancer detection but has poor specificity and prognostic accuracy. The result of this ambiguity is that, amongst men who have undergone initial therapy, it is uncertain who will relapse with recurrent aggressive disease [5, 6]. These ambiguities are clinically relevant because men who experience treatment failure have a significantly increased risk of dying of CaP [7]. A similar difficulty exists over the accurate identification of indolent disease, in which either radical surgery or high dose radiation treatments could be deferred [1, 2].

MicroRNAs (miRNAs) hold considerable promise to be exploited as highly accurate and functional prognostic serum markers of CaP stages and drug responses [8-18]. They can encapsulate events within the tumor micro-environment and thereby overcome the limitations of inaccurate tumor sampling at biopsy. From a biostatistical perspective, given there are fewer miRNA than protein coding mRNA, genome-wide coverage is more readily achieved and avoids the statistical penalties typically associated mRNA genome wide testing [19].

Despite encouraging results from analyzing miRNA expression to define later stages of CaP [20, 21], it has yet to be demonstrated that miRNAs can be leveraged in combination with non-invasive clinical measures (e.g. PSA) at an early disease stage to identify those cancer patients who will rapidly recur after treatment. The goal of the current study was to find miRNA expression patterns in men with localized CaP that associate with the risk of early treatment failure defined by biochemical progression soon after RP and/or can be exploited to predict progression better than pathological and/or clinical parameters alone [22].

**Figure 1:**
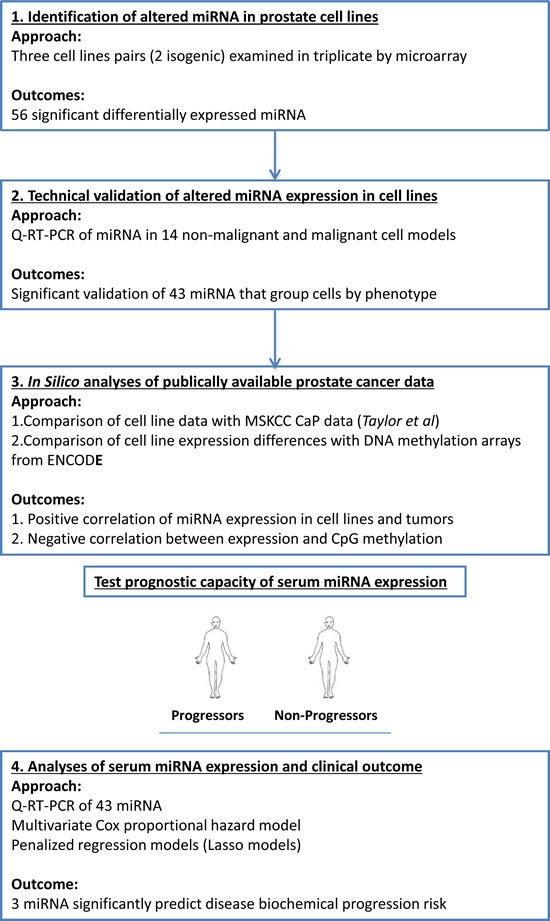
The workflow designed to identify and test the prognostic capacity of miRNA in the serum of patients with CaP

To meet this challenge we used a combination of cell line approaches and *in silico* analyses of publically available data derived from CaP tumors to identify miRNA associated with different CaP stages (Figure 1). The capacity of these miRNA to distinguish progression risks and treatment responses were subsequently measured in serum samples from CaP patients. Principally, we focused on serum collected prior to surgery, in a clinical cohort of men with localized CaP who underwent RP, with the overall goal of defining miRNA expression patterns associated with the risk of biochemical progression. Three miRNAs (miR-103, 125b and 222) were identified that, at the time of surgery, associated with the subsequent risk of biochemical progression. When combined with PSA levels, these miRNA generated strong predictive models that identified men who would experience biochemical progression. Critically, these models, derived from non-invasive measurements, were as effective at predicting biochemical progression as the tumor grade derived from the pathological examination of the surgically removed tumor. These findings suggest that serum miRNA expression patterns in combination with PSA levels can be exploited to stratify patients for optimal therapeutic response, even prior to surgery.

## 2 RESULTS

### 2.1 Identification of miRNA expression patterns in non-malignant and malignant prostate cell lines

**Figure 2:**
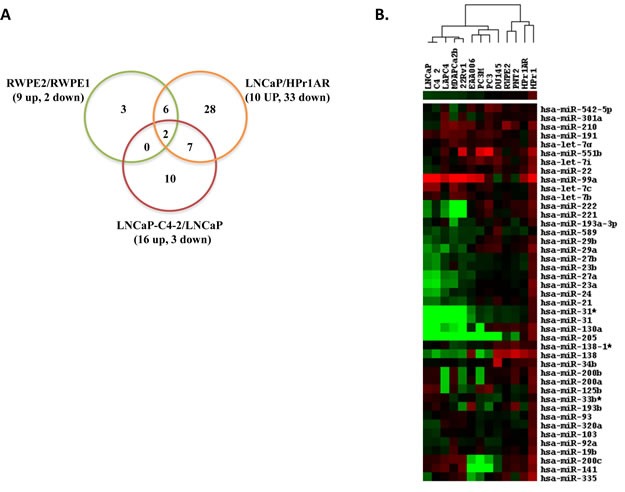
Identification of differentially expressed miRNAs by microarray and Q-RT-PCR in CaP cell lines A. The number of differentially up and down regulated miRNAs identified in three cell line pairs representing different clinical stages of CaP. Non-malignant RWPE-1 and their isogenic RAS-transformed counterpart RWPE-2; non-malignant HPr1-AR and LNCaP; and LNCaP and their isogenic ADT recurrent variant LNCaP-C4-2 cells using Exiqon miRNA microarray. Cells were grown to mid-exponential density and RNA isolated prior to microarray analyses. All experiments were undertaken in triplicate. B. 43 miRNA were measured by Q-RT-PCR in a panel of 14 non-malignant and malignant cell lines. Expression analysis was performed using Exiqon miRNA specific primers. The relative expression of miRNAs in different cell lines was calculated compared to RWPE1 cell line. Heat map shows log2 fold change values (green representing downregulation, red representing upregulation relative to RWPE1).

The purpose of these experiments was to identify altered expression patterns of miRNAs that reflect different CaP stages, by agnostically analyzing miRNA expression levels in cell line models; specifically RWPE-1 vs RWPE-2 [33]; HPr1AR vs LNCaP [34]; LNCaP vs LNCaPC4-2 [35]. MiRNA microarray analyses of these three pairs of cells identified a total of 56 miRNAs as significantly altered (1.5 fold, *p*<0.05) (Figure 2A and Table 1). Comparison between RWPE-1 vs RWPE-2 (encompassing CaP initiation) identified 11 significant changes, and of these miRNAs, 9 were up-regulated and 2 were down-regulated in RWPE-2. Comparison between HPr1-AR vs LNCaP (encompassing CaP initiation & progression) identified 43 significant changes of which 33 miRNAs were down-regulated and 10 miRNAs were up-regulated in LNCaP. LNCaP vs LNCaP-C4-2 (encompassing the ADT-CaP phenotype) revealed 16 miRNAs that were significantly up-regulated and 3 miRNAs were down-regulated in LNCaP-C4-2. HPr1AR vs LNCaP, and LNCaP vs LNCaP-C4-2 comparisons revealed nine common miRNAs (Figure 2A and Table 1). Out of 11 significant changes in RWPE-1 vs RWPE-2, 8 miRNAs were common with HPr1AR vs LNCaP and two with LNCaP vs LNCaP C4-2. Two miRNAs were differentially regulated across the three comparisons (miR-335 and miR-31, Figure 2A and Table 1). Collectively, these findings suggest there are common and unique miRNA expression patterns for different stages of CaP.

**Table 1:** miRNA expression patterns identified in microarray analysis and validated by Q-RT-PCR in cell lines and CaP serum samples RNA was isolated from the indicated cell lines and human tumor samples using Trizol and from serum samples using Qiagen miRNeasy kit with Exiqon recommended modifications. Microarray analysis was performed with Exiqon miRNA 5th generation expression arrays using single color hybridization in biological triplicates for each cell line. 384-well plates Q-RT-PCR analysis was used with Exiqon miRNA specific primers. All miRNAs with missing values (ct>38) were removed. Samples were then normalized using the overall mean miRNA expression value. MiRNA differentially expressed in microarray analysis and validated by Q-RT-PCR are shown in bold letters with asterisk mark. (≥1.5 fold change and *p*≤0.05). ND, not detected, failed in Q-RT-PCR; NA, primers to validate not available

miRNA	Prostate cell line miR studies						miR Serum study	
	RWPE-2 vs RWFE-1 (Log_2_ fold change)		LNCaP vs HPrAR-1 (Log_2_ fold change)		LNCaP-C4-2 vs LNCaP (Log_2_ fold change)		Non-progressors (N=62)	Progressor s(N=31)
	Micro-array	Q-PCR	Micro-array	Q-PCR	Micro-array	Q-PCR	Average dCT	Average dCT
hsa-miR-210	1.311*	3.527*	0.006	-1.015*	-0.055	0.078	-3.395	-3.372
hsa-miR-200c	0.920*	0.973*	0.244	0.013	0.134	0.112	-6.067	-6.369
hsa-miR-141	0.906*	0.895*	0.183	-0.155	0.629	0.665	-7.635	-7.813
hsa-miR-22	0.879*	**0.508**	-1.397*	-2.59*	-0.497	0.425	-1.506	-1.535
hsa-miR-130a has-let-7i hsa-miR-335 hsa-miR-138	0.746* 0.641* 1.001* 1.513*	1.248* 1.075* 1.798* 4.027*	-1.669* -0.605* -1.522* -1.805*	-8.101* -1.847* -4.53* -7.01*	0.012 0.298 0.656* -0.143	0.021 0.927* 2.42* -1.799*	-3.253 0.072 -4.813 ND	-3.316 -0.262 -4.780 ND
hsa-miR-31	-0.679*	-1.185*	-3.757*	-10.348*	0.58*	0.478	-7.564	-7.624
hsa-miR-205	-1.032*	-1.365*	-5.941*	-16.521*	0.035	-0.407	-6.228	-6.110
has-miR-222	0.136	0.430	-2.397*	-4.54*	1.125*	2.543*	-0.977	-0.668
hsa-miR-221 hsa-miR-193a-3p hsa-miR-138-l* hsa-miR-29b	0.005 -0.316 -0.266 0.069	-0.482 0.223 1.55* -0.345	-1.026* -0.887* -0.664* -2.5*	-4.673* -1.2* -1.527* -3.22*	0.529 0.904* 0.96* 0.818	2.64* 0.818* 0.215 0.998*	-0.031 ND ND -4.287	-0.230 ND ND -4.490
hsa-miR-320a	0.239	0.080	-0.596*	-0.742*	0.292	-0.015	1.942	1.955
hsa-miR-301a	0.328	0.452	-0.624*	-1.137*	0.186	0.168	-4.326	-4.354
hsa-miR-103	0.136	0.272	-0.67*	-1.118*	-0.068	-0.218	2.144	1.821
hsa-miR-92a hsa-miR-27a hsa-miR-19b hsa-rruR-27b	0.060 -0.046 0.167 -0.086	0.008 -0.222 0.325 -0.063	-0.808* -1.016* -1.024* -1.133*	-0.817* -5.008* -0.632* -2.34*	0.537 -0.283 0.374 -0.156	0.052 -0.312 0.342 -0.508	3.855 -0.257 3.001 -1.960	3.690 0.029 3.031 -1.623
hsa-miR-21	0.040	-0.038	-1.242*	-2.765*	-0.113	-0.077	1.992	1.855
hsa-miR-24	0.094	-0.040	-2.724*	-4.112*	-0.085	-0.312	2.017	1.988
hsa-miR-29a	0.123	0.098	-2.885*	-4.735*	0.532	1.337*	-1.706	-1.716
hsa-miR-23b	0.508	-0.102	-3.227*	-2.033*	-0.292	-0.608*	-0.874	-0.839
hsa-miR-31* hsa-miR-23a hsa-miR-99a	-0.479 0.525 0.049	-1.125* 0.097 0.908*	-1.86* -3.797* 2.994*	-10.237* -5.79* 8.115*	0.040 0.017 -0.175	-0.407 -0.257 -0.175	ND 1.474 -3.961	ND 1.450 -3.721
hsa-miR-125b	0.403	0.023	2.421*	2.92*	-0.209	-0.278	-3.614	-3.081
has-let-7c	0.106	-0.062	2.019*	3.943*	0.088	-0.022	-4.379	-4.417
hsa-miR-191	0.241	0.467	1.466*	2.22*	-0.397	-0.483	-0.474	-0.589
has-let-7b has-let-7g hsa-miR-200a hsa-miR-200b hsa-miR-33b*	0.521 0.296 0.399 0.500 0.029	0.533 0.623* 0.363 0.703* 0.092	1.354* 0.765* 0.759* 0.647* 0.633*	1.212* 0.713* 1.038* 0.517 1.442*	0.136 -0.420 -0.190 -0.059 -0.445	0.61* -0.188 -0.093 0.067 -0.268	2.244 1.127 ND ND ND	2.266 0.909 ND ND ND
hsa-miR-542-5p	0.204	0.517	0.360	-0.705	-0.66*	-0.848	-7.351	-7.262
hsa-miR-93	-0.114	-0.443	-0225	-0.418	0.722*	0.513	2.481	2.318
hsa-miR-34b hsa-miR-589 hsa-miR-551b hsa-miR-193b	-0.027 0.109 -0.128 0.400	0.019 -0.988 -0.443 0.697*	-0.174 -0368 0.086 0.434	-1.776* -0.210 -1.614* 1.762*	0.68* 0.652* 0.608* 0.61*	0.052 -0.647 -0.296 0.682*	ND ND -7.315 -6.446	ND ND -7.582 -6.187
hsa-miR-488*	-0.099	ND	0.198	ND	0.744*	ND		
hsa-miR-634	-0.167	ND	-0264	ND	0.647*	ND		
hsa-miR-765	0.040	ND	-0.816*	ND	0.773*	ND		
hsa-miR-129-3p	0.034	ND	-0.607*	ND	-0.064	ND		
hsa-miR-494	0.165	ND	-0.585*	ND	0.527	ND		
hsa-miR-32*	0.080	NA	-0.407	NA	0.605*	NA		
hsa-miR-2114	-0.029	NA	0.985*	NA	-0.989*	NA		
hsa-miR-585	0.090	NA	0.454	NA	-0.637*	NA		

The 56 miRNAs that were identified to be differentially expressed by the microarray approach were selected for subsequent validation by Q-RT-PCR, using Exiqon LNA™ miRNA specific primers. Validated PCR primers were unavailable for 8 miRNAs, and 5 miRNAs failed QC and were excluded from the analysis. Thus, 43 miRNAs were tested for validation that included 10 miRNAs associated with RWPE-1 vs RWPE-2, 34 in HPr1AR vs LNCaP and 11 in LNCaP vs LNCaP C4-2 (Table 1). Q-RT-PCR validation of miRNA expression changes in the cell line pairs showed strong concordance with the patterns observed in the microarray data; 9 of 10 in RWPE-2, 33 of 34 in LNCaP and 4 of 11 in LNCaP-C4-2 showed significant changes similar to microarray data (>=1.5 fold, *p*<0.05, Table 1). Additionally, 17 other miRNAs in these three cell line combinations that failed to reach a significance level in microarray analysis revealed modest but significant differential regulation in Q-RT-PCR analysis, for example miR-200b in RWPE-2, miR-193b in RWPE-2 and LNCaP and miR-29a in LNCaP C4-2 (Table 1).

Subsequently, these 43 miRNAs were measured in a panel of 14 CaP cell models, which included the original 6 cell lines used for the microarray analyses. 12 of these cell lines were derived from European American (EA) and two from African American (AA) patients. Relative miRNA expression in these cell lines was calculated compared to non-malignant RWPE-1 cells. The expression patterns of these 43 miRNAs, detected by Q-PCR in the 14 cell line panel, are represented in Figure 2B. The EA cell lines with similar clinical features (e.g. non-malignant, androgen sensitive CaP and ADT-RCaP) showed related expression changes and, using hierarchical clustering, were grouped together (Figure 2B). The AA ADT-RCaP model (MDA PCa 2b) was comparable to the EA ADT-RCaP model (LNCaP C4-2). However, the AA androgen-sensitive model (E006AA) grouped with the metastatic EA cell lines (e.g. PC-3 M). These data suggest that altered miRNA expression magnitude and direction can distinguish between cell lines from different stages of CaP, including those derived from men of different race.

### 2.2 *In silico* analyses of miRNA expression in human CaP

Next we tested whether differentially expressed miRNA patterns, identified and validated through the prostate cell line analyses, reflect miRNA expression in human CaP tumors. For this analysis we interrogated publically-available miRNA expression data in primary tumors from the MSKCC cohort [28]. Only CaP patients reporting as EA non-hispanics and who underwent RP or laproscopic RP (LRP) were used for analysis. Relative expression of miRNAs in primary prostate tumors (N=78) from EA men were calculated compared to normal prostate tissue from EA men (N=25). Normalized log_2_ expression data for miRNAs were downloaded from the cBioPortal and compared to the RWPE-1 vs RWPE-2, and HPr1AR vs LNCaP microarray data.

**Figure 3:**
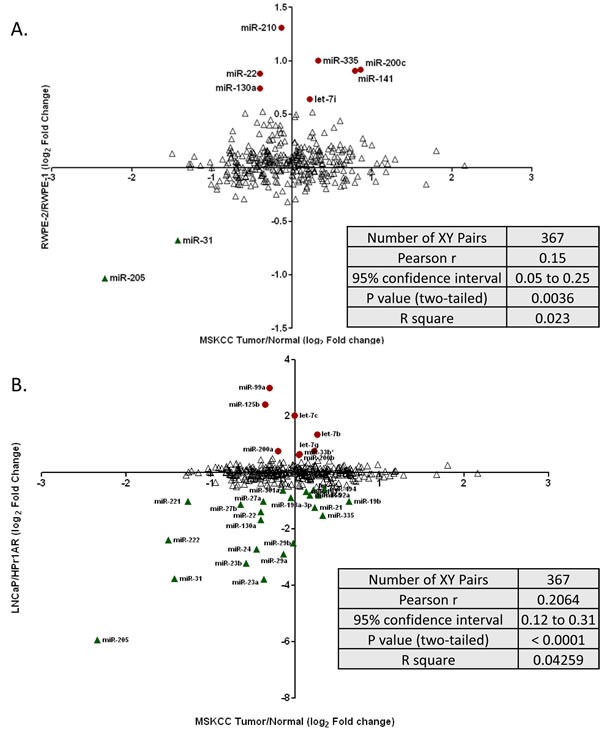
Scatter plot showing the correlation of miRNA expression between prostate cell lines and publically available CaP tumors Log 2 fold change of miRNA expression was calculated for 78 primary CaP tumors compared to 25 matched normal tissue from Taylor et al study (MSKCC cohort) and compared to the log 2 fold change analyses obtained through cell line comparisons. A. miRNA expression primary in CaP tumors compared to RWPE2/RWPE1 cells. B. miRNA expression in primary CaP tumors compared to LNCaP/HPr1AR cells. Up regulated miRNAs in cell lines are shown as red circles, donwregulated miRNAs in cell lines are shown as green triangles. Red and green symbols show significant correlation between the data sets. Blue symbols are miRNA that were not significantly correlated.

367 miRNA were in common between the two microarray platforms and their expression patterns are represented as scatter plots in Figure 3. These analyses revealed a significant correlation between expression patterns identified by the cell line and primary tumor analyses. For example, the differentially expressed miRNAs between RWPE-2 vs RWPE-1 and the primary tumor vs. adjacent normal comparison revealed a significant correlation of both up (e.g. miR-141) and down (e.g. miR-31) regulated miRNA (p<0.0036). Similar significant findings (p<0.0001) were identified by comparison of LNCaP cells compared to HPr1AR and miRNA changes in primary tumors (e.g. miR-222). This *in-silico* validation suggested that miRNA expression patterns identified through cell line analysis are comparable to expression in human CaP tumors (Figure 3).

### 2.3 Serum miRNA levels at the time of RP significantly associate with the risk of biochemical progression

**Figure 4:**
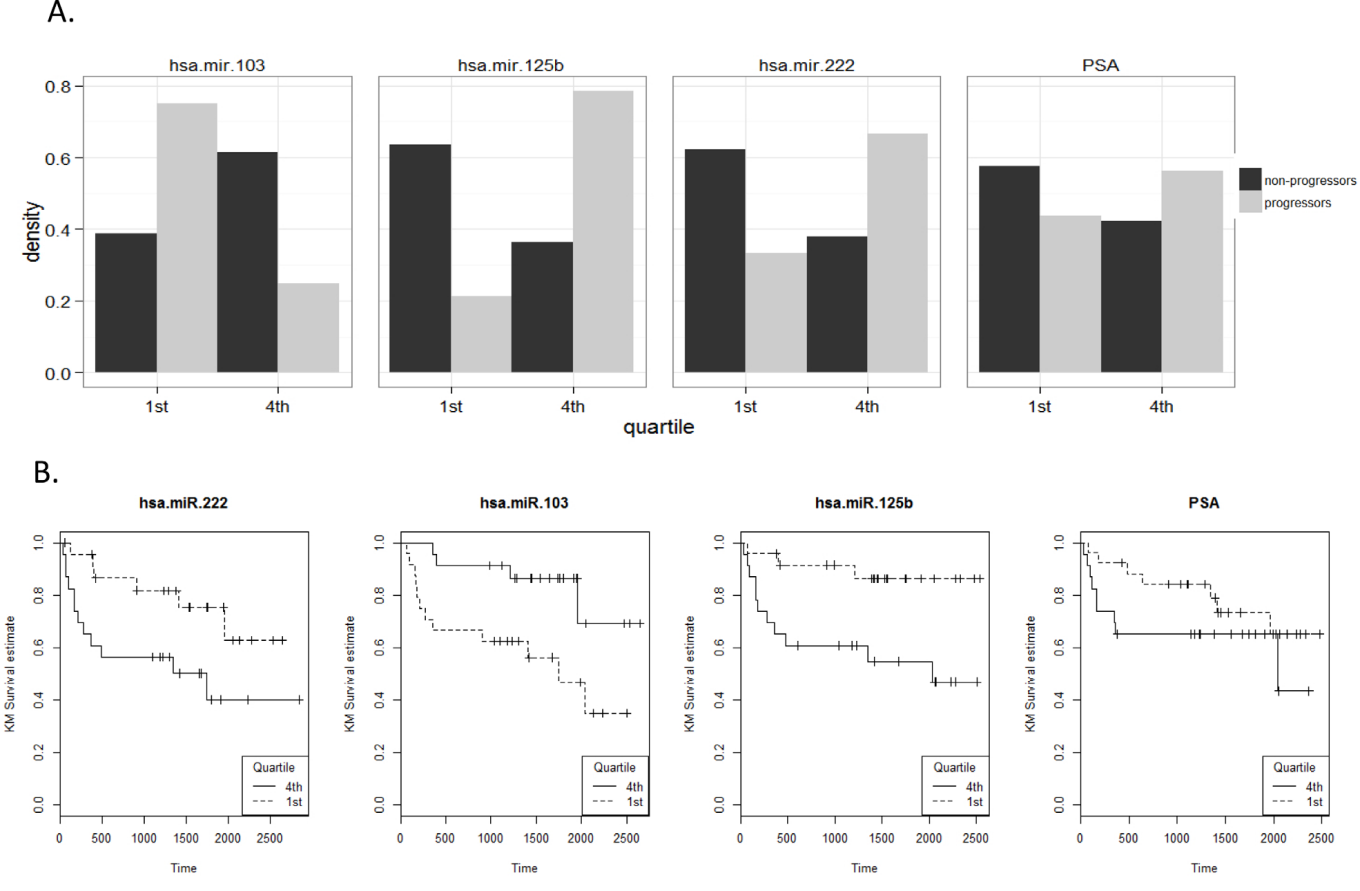
A. Distribution of miRNA serum expression values within the RPCI cohort MiRNA expression levels were divided into quartiles for the entire cohort. For each quartile the proportion of the total number of samples (y axis) is shown for progressors and non-progressors. B. Time to biochemical progression analyses for miRNA. Quartile analyses of these miRNA (first and fourth) remain significant at a similar magnitude, with quartile four values for miR-222 and miR-125b showing significant increase in hazard of biochemical progression compared to quartile one; CaP patients with miR-103 values in quartile one showed a decrease risk of progression compared to men in quartile four. Time is measured in days from surgery.

The ability of the 43 miRNAs, identified through cell line and *in silico* analyses, to predict the risk biochemical progression was tested in a well-defined clinical cohort of 99 men with primary CaP, who were frequency matched on age and Gleason grade sum. Six serum samples failed Q-PCR quality control and were removed from subsequent analysis; the patient characteristics of these 93 samples are described in Table 2 (62 patients who did not progress during follow-up and 31 patients who progressed). 9 miRNAs were undetected in serum (ct > 38) and were removed from the further analysis. Of the 34 miRNAs remaining that were serum detectable, 3 were found to be differentially expressed in the serum of men who progressed compared to those who did not (miR-103, miR-125b, mir-222). The proportion of men who progressed is shown for these three miRNAs serum expression values and PSA quartiles in Figure 4A. The proportion of progressors is significantly different between first and fourth miRNA quartile (*p*<0.0001) and with PSA level (*p*<0.001).

**Table 2:** Patients characteristics used in case-cohort study of miRNA association with risk of biochemical progression All serum samples were collected with IRB approval at RPCI. Patient serum and demographic, epidemiologic, clinical and pathology data were collected through the Data Bank and BioRepository (DBBR), a shared resource at RPCI. All blood specimens were collected from newly diagnosed men with CaP, prior to any treatment. All patients had at least 3 years of follow-up data. Biochemical progression was defined by serum PSA of 0.2 ng/mL or greater (obtained 6 weeks – 3 months postoperatively), with a second confirmatory level of PSA greater than 0.2 ng/mL (N=31, classified as progressors), all other men were classified as non-progressors (N=62). Non-Progressors were selected from a larger group of CaP patients with equivalent follow-up and frequency matched on age and Gleason grade sum using a bootstraping procedure. *Average follow-up time for non-progressors

Variables	Progressors	Non-progressors
N	31	62
Age at diagnosis	59.2	59.8
PSA at Surgery	10.7	6.3
Average Time to Progression (days)*	624.6	1634.6
Gleason Grade Sum		
6	3	7
7	20	44
8	2	2
9	6	9

**Table 3:** Hazard of biochemical progression Multivariate Cox proportional hazard models were used to determine the miRNA, pathological and clinical features that were significantly associated with hazard of biochemical recurrence.

Model	HR	Lower 95% CI	Upper 95% CI	Model p-value
PSA	1.05	1.02	1.08	0.001
Gleason Grade Sum	1.22	0.82	1.80	0.32
Age at Diagnosis	0.99	0.93	1.06	0.84
BMI	1.05	0.97	1.12	0.19
hsa.miR.222	2.80	1.29	6.10	0.009
hsa.miR.103	0.41	0.21	0.79	0.008
hsa.miR.125b	1.79	1.10	2.91	0.018
hsa.miR.222, adjusted for PSA	2.60	1.17	5.76	0.0004
hsa.miR.103, adjusted for PSA	0.41	0.20	0.82	0.0002
hsa.miR.125b, adjusted for PSA	1.78	1.08	2.95	0.0006

We then measured the hazard of biochemical progression attributable to clinical and pathological characteristics, as well as serum expression for each of these three miRNA (normalized ct values) (Table 3). We found a modest but significant association with PSA such that for each unit increase in PSA at diagnosis there was a 5% increase in hazard of biochemical progression. Given the frequency-matching schema, in which patients in the two groups were matched based on Gleason grade sum and age, it is not surprising that Gleason grade sum was insignificant as was age and BMI at time of diagnosis. The hazard associated with each unit increase in normalized ct value of miR-222 was 2.8 (1.3 - 6.1 95% CI) and for miR-125b it was 1.8 (1.1 - 2.9 95% CI). In contrast, each unit decrease in miR-103 associated with an almost 2.5 fold decrease in hazard of biochemical progression (HR=0.41 with 0.2-0.8 95% CI, Table 3).

To make the clinical data more suitable for analyses, we separated it into discrete categories (quartiles). Again, not surprisingly, quartile analyses of these miRNAs (first and fourth) remain significant at a similar magnitude. Quartile four values for miR-222 and miR-125b revealed a significant increase in hazard of biochemical progression compared to quartile one; CaP patients with miR-103 values in quartile one showed a decrease risk of progression compared to men in quartile four (Figure 4B). These three miRNA were highly correlated with one another: miR-222 and miR-125b positively correlated (*ρ* =0.74); miR-222 and miR-103 negatively correlated (*ρ* =−0.37), as were miR-125b and miR-103 (*ρ* =−0.56).

Importantly, the miRNA values and clinical variables represent different contributions to hazard of biochemical progression, as reflected by the fact that no significant correlations were found between PSA, Gleason score, age, BMI and any of the three significant miRNA. The best fitting hazard model included both miR-103 and PSA (*p*<0.0002), although all three miRNAs when combined with PSA were of similar significance levels (Table 3).

We also measured serum expression of the 34 miRNA used above in two small cohorts of men who had progressed after RP and were undergoing treatments for non-localized metastatic stage. Specifically, these were men with hormone sensitive prostate cancer (HSPC, N=17) treated with anti-androgen therapy, and men with ADT-RCaP being treated with chemotherapy (N=14). In the HSPC group, 11 miRNA differed between time points, the most significant, miR-320a (log_2_ fold change =-0.92, p<.0001, Supplementary Table 1). Of the miRNAs identified as differentially expressed in men with localized disease, miR-222 was also differentially expressed between the time points (log_2_ fold change=-0.37, *p*<.04). In the ADT-RCaP group, we measured association of miRNA with hazard of death (5/14 patients died). While none of these 3 miRNA were significant alone, a model including PSA and miR-103 was suggestive of statistical significance (*p*<0.06), although due to the small sample size the hazard ratio confidence intervals were wide (HR=2.65 with a 95% CI of 0.55-15.5). These findings also added confidence to the identification of these miRNA in the RP cohort as being associated with disease progression.

### 2.4 Correlation of miRNA expression in tumor and serum

Finally, we also measured expression of the 34 miRNAs in matched serum and primary tumor from a limited subset of ten CaP patients from RPCI whose serum samples were analyzed as part of the biochemical progression analyses. All three miRNA, miR-103, mir-125b and miR-222, showed relatively similar expression levels in the tumor and the serum in each of the ten patients suggesting that, for these miRNA at least, the levels detected in serum accurately reflect the expression of miRNA in their tumor counterparts. This was not universal for all miRNA.

**Figure 5:**
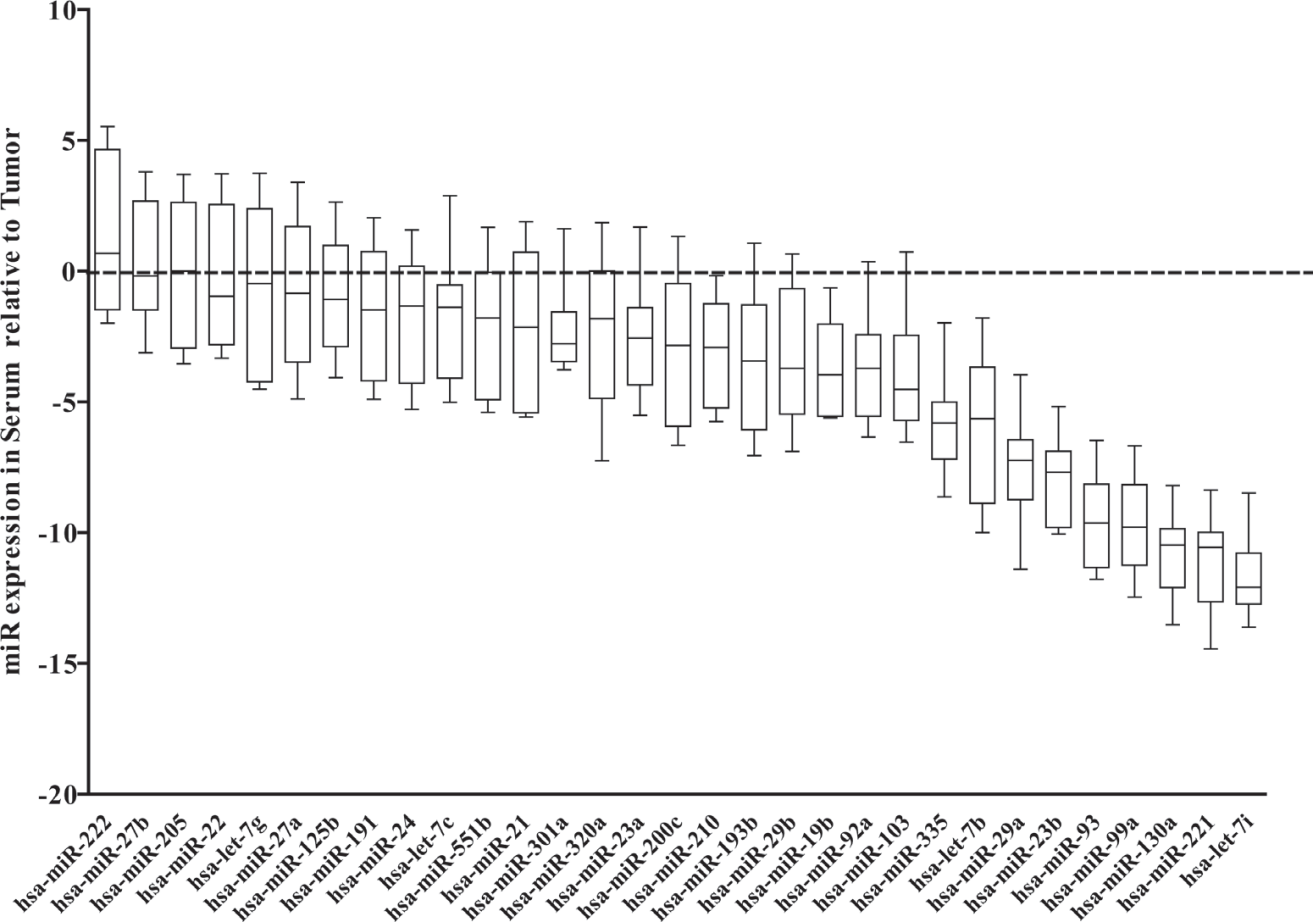
miRNA expression in matched tumor and serum samples Expression of miRNA was analyzed in paired serum and tumor samples from 10 CaP patients. miRNA expression was performed using Exiqon miRNA specific assay by Q-RT-PCR. Raw ct values were mean centric normalized then difference between serum and tumor samples were calculated (dCT_Serum_-dCT_Tumor_).

### 2.5 Generating predictive models of biochemical progression

**Figure 6:**
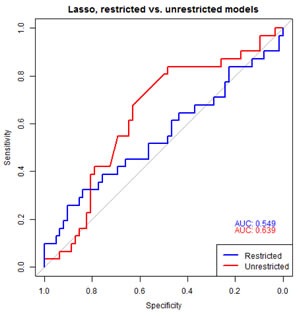
Predictive models of biochemical progression To assess predictive value of the significant miRNAs, penalized regression analyses was performed including all miRNA, PSA, age and grade. The AUC for the restricted lasso model containing PSA, age and grade was 0.52 (95% CI 0.38-0.64, Blue line). The unrestricted lasso model contained covariates PSA, miR-103, miR-125b, and miR-222 with AUC 0.67(95% CI, 0.55-0.79, Red line).

To assess the predictive value of the miRNA for biochemical progression, we undertook penalized regression analyses with leave one out cross validation with all 34 serum detected miRNAs between the two groups, PSA, age and pathological grade [31, 32]. The AUC for the restricted lasso model containing PSA, age and pathological grade was 0.55 (95% CI, 0.38-0.64, blue line in Figure 6). The unrestricted lasso model again, considering all miRNA and clinical parameters, contained covariates PSA, and three miRNAs out of 34 tested (miR-103, miR-125b, and miR-222) with AUC 0.64 (95% CI, 0.55-0.79, Red line in Figure 6).

This finding underscores the discovery of significant changes in expression of these three miRNA between men who progressed compared to those with stable disease. Although there was a difference between the predictive value of the AUC, a bootstrap test of significance comparing restricted versus unrestricted lasso model indicated it was not significant at the 0.05 level (*p*=0.25). Similar AUC estimates were obtained using two separate approaches [31, 32]. Together, these analyses revealed that serum expression levels of three miRNAs in conjunction with PSA are equally able to predict men who are at risk of experiencing treatment failure as the analyses of pathological parameters obtained after the radical procedure of surgery.

## 3 DISUSSION

There is considerable enthusiasm to exploit serum miRNA expression in diagnosis and prognosis in cancer. In CaP, miRNA patterns have been associated with extremes of disease progression, for example comparing either tumor or metastatic disease [36, 37] to non-malignant conditions [18, 28, 38-40]; or comparing prostate cancer at the time of surgery to benign prostatic hyperplasia [37, 41, 42]; or comparing different tumor grades [43]. Although there is a rapidly expanding literature on CaP miRNA serum expression patterns (Supplementary Table 2), the current study is significant for several reasons. Firstly, the current approach is the first study to establish that miRNA in combination with *non-invasive* clinical parameters could be used to predict early CaP progression without knowledge of the underlying tumor grade. Secondly, the use of serum miRNA is as effective, and suggestive of being better, than analyses of the pathological parameters alone that are recovered following surgery.

We began with a thorough analyses of cell line models, reasoning that cell lines are more pure cell populations compared to primary tissue, and therefore would yield more accurate differential miRNA expression patterns to carry forward to a human cohort. Microarray and Q-RT-PCR approaches in a 14 prostate cell line panel supported the concept that the expression patterns of 43 miRNA could accurately distinguish of non-malignant, androgen sensitive and ADT-RCaP cell types. This miRNA panel includes miRNAs previously associated with resistance to chemotherapies (miR-130a) [44] and poor progression free survival (miR-200c and miR-141) in CaP [45]. These also included miRNAs associated with regulation of proliferation, metastasis and poor progression free survival [46-48]. Pathway enrichment analyses [29] of these differentially expressed miRNA identified significant targeting of TGF-β and WNT networks (reviewed in[49, 50]) (Supplementary Table 3). For example, the comparison of RWPE-2 to RWPE-1 identified 3 miRNAs (e.g. miR138, 200c and let-7i) that targeted thirty-four genes on the TGF-β pathway, including *TGBR1* and *SMAD4* [51, 52]. Interestingly, loss of Smad4, a downstream regulator of TGF-β, drives invasive CaP in co-operation with *Pten* depletion [53]. The cell lines represent different stages of disease progression and it is interesting to note that the miRNA expression patterns continue to change between the different cell types and disease states; miR-138 was up-regulated in RWPE-2 compared to RWPE-1, but down-regulated in the androgen sensitive cancer cell lines (Table 1). This re-enforces the concept that unique and shared miRNA expression patterns (both in terms of magnitude and direction) may precisely reflect CaP disease states, and determine the risk of CaP progression.

Interestingly, epigenetic events are implicated in the control of miRNA expression [54], (reviewed in[55]). To examine the basis for the altered miRNA expression we considered the impact of DNA CpG methylation. We mined ENCODE data sets of genome wide DNA methylation patterns in LNCaP compared to PrEC non-malignant prostate cells to examine the distribution of methylation at CpG sites within ±5kb of the 35 miRNA found in the current study to be altered in LNCaP as compared to RWPE-1 cells. 23 miRNA displayed an inverse relationship between DNA CpG methylation status and miRNA expression levels, suggesting that CpG methylation impacts the expression of miRNA in CaP to a significant extent (Supplementary Figure 1, Supplementary Table 4) and may be associated with tumor initiation.

Of the 43 miRNA identified in the cell line comparison only 34 were adequately detected in the serum of CaP patients, and of these 3 miRNAs (miR-103, miR-125b, miR-222) were differentially expressed at the time of surgery between patients who were otherwise indistinguishable in terms of age, PSA levels, tumor stage derived from biopsy material but diverged in terms of displaying either stable disease or biochemical progression. Models were constructed to measure the relationship between serum miRNA expression, the hazard of progression and the ability of the miRNA to predict progression alone and in conjunction with PSA, stage, grade and BMI. Irrespective of the modeling approach, our results consistently identified that differential expression of miR-103, miR-125b and miR-222 predicted biochemical progression. Of these, miR-222 [14, 56-61] and to a lesser extent miR-125b [21, 41, 42, 59, 62] appear to play an important role in prostate biology, whereas to-date miR-103 has not been associated in CaP, although has been associated with other cancers [63-65].

MiR-222 is transcribed as a miR cluster with miR-221 from chromosome X11p.3. Felli et al. (2005) first described miR221/-222 as integral regulators of *in vitro* erythropoiesis[66] and deregulated expression of the cluster is implicated in several cancers [67-75] suggesting that these miRs have oncogenic potential. In general, oncogenic properties of miR-221 and miR-222 are attributed to their control of the cyclin dependent kinase (Cdk) inhibitors p27KIP1 and p57KIP2, and thus control of G1 to S phase transition[56, 57] PI3K and PTEN signaling[14] and other targets implicated in malignant transformation, including CX43[68], RECK[71], and ERα[76, 77].

Tissue miR-222 expression was also recently found to predict progression free and overall survival in gastric carcinoma patients[73], and miR-222 detection in urine was shown to detect bladder cancer with high accuracy, thus providing a potential noninvasive diagnostic biomarker in this setting, as well as predicting for recurrence, progression, and overall survival[75]. In CaP, Lin et al. (2011) observed that tumors (Gleason grade ≥ 7) had elevated expression of miR-221 and miR-222 compared to less aggressive tumor tissues (Gleason grade < 7), further suggesting an oncogenic role of these miRs in CaP development[58]. Interestingly, an assessment of 40 early stage (T2a/b) prostatectomy specimens revealed loss of miR221/-222 expression in microdissected malignant tissue compared to non-involved tissue controls[59], perhaps indicating that expression levels are differentially modulated at divergent stages of disease and evidence also suggests that miR221/-222 is involved in ADT-RCaP[60]. Most recently, a miRnome wide scan found miR-222 to be the most significantly differentially expressed miR when comparing 20 matched pairs of microdissected tissue samples of prostate cancer and non-tumor tissue[78].

MiR-125b was also elevated in men with progressive disease in the current study although the function is ill-defined as it has been reported to function as oncogene or tumor-suppressor gene in different cancer types or cell lines[79]. One of the earliest studies on miR in CaP identified six miR including miR-125b and miR-143 to be upregulated in metastatic CaP serum samples as compared to normal individuals [17]. Expression of miR-125b in serum of CaP patients is reported to be upregulated as compared to normal controls[21] whereas other studies reported it to be downregulated in CaP as compared to normal or BPH samples[41, 42, 59]. MiR-125b regulates cell proliferation in prostate cancer cell lines cells[62], and it has suggested to be upregulated by androgen signaling[80]. Functionally in CaP, miR-125b has been reported to target *BAK1*[80] (a pro-apoptotic member of the BCL-2 gene family) and *EIF4EBP1*[41] (Eukaryotic translation initiation factor 4E-binding protein 1, a gene that encodes one member of a family of translation repressors proteins) and the transcriptional co-repressors NCOR2/SMRT [81].

Our finding of reduced miR-103 in the serum of men whose disease progressed and association with hazard of progression is a novel one in CaP. The aberrant overexpression of miR-103 has been reported in many cancers including endometrial[63], bladder[64] and breast cancers[65]. Increased expression of miR-103 was associated with metastasis and poor outcome in breast cancer[65]. Functionally miR-103 targets Dicer and thus can attenuate miR biosynthesis. MiR-103 has also been shown to regulate cancer metastasis by regulating EMT and inhibition of miR-103 opposes migration and metastasis[65].

Tumor-serum correlation analysis of miRNA expression in a subset of these patients revealed variations in detectible expression suggesting parallel processes which govern serum miRNA detection [82-84] including active miRNA export where the serum levels were high, and passive release for miRNA with lower and poor correlations between serum and tumor expression levels. For the 9 miRNA that were undetectable in serum, despite robust expression in tumors, it suggests that there are differential secretion of miRNA, or selective degradation in the serum, or that secreted miRNA profile reflects a more complex interaction of different cell types within the tumor micro-environment.

The potential for altered tumor and serum relationships was also highlighted in our preliminary validation studies. In this case we examined the association of miR-103, miR-125b and miR-222 with biochemical progression in the 78 white non-hispanic patients from the MSKCC data set. Patients were separated into those with high or low miRNA expression using a median expression level threshold, and proportions of each subgroup to experience biochemical progression over time were calculated as Kaplan-Meier curves (Supplementary Figure 2). MiR-125b expression associated with time to biochemical progression (*p*<0.06) in this cohort. Interestingly, low CaP tumor expression of miR-125b predicts increased overall probability of progression, and is opposite of what we observed in the serum of CaP patient, where elevated expression significantly predicts increased probability of biochemical progression. However, active secretion of miR-125b has been reported in serum upon castration in mice and in extracellular media upon androgen blockade in LNCaP cells with simultaneous loss of expression in cellular compartments [81], most likely downstream of AR actions. Therefore these trends may indicate that patients with the most aggressive disease have most active export from the tumor (and therefore lowest tumor levels) and the highest levels of miR-125b in the serum as a result. Analysis of the expression of miR-125b in the matched tumor serum samples was supportive of this relationship also. The expression levels in the matched tumor and serum samples was suggestive of lower tumor expression and higher serum levels in the progressors compared to the non-progressors (Supplementary Figure 2).

Recently, Selth et al., [13] demonstrated there may be evidence for association of serum expression of miR-146-3p and miR-194 with reduced time to biochemical progression. Specifically, the authors suggested that high levels of miR-146b-3p expression, pre-operative PSA and seminal vesicle invasion, obtained following invasive surgery, were predictors of a reduced recurrence free interval. Furthermore, this miRNA was either very low or not detectable in greater than one half the samples in either of the two publically available CaP tumor cohorts examined (MSKCC or Erasmus) precluding further evaluation. When considering only miR-194 expression levels values, quartile one versus four, there was a significant association with time to biochemical progression in the MSKCC and the Selth et al. cohort, respectively.

However, miR-194 expression did not show significant association with time to biochemical progression in the Erasmus cohort. Interestingly, in the MSKCC cohort the CaP patients with metastases, who comprise a majority of the low miR-194 group, drove the statistical differences for these comparisons. The prominent role of metastatic cancer (versus primary) implies that miR-194 could be a marker of other events such as general inflammation rather than specifically biochemical progression. Furthermore, the unique contribution of the miRNA expression to biochemical progression was hard to establish, in that both miR-194 and miR-146-3p were weakly correlated with PSA and highly correlated with age and surgical margins, respectively; the later association perhaps explaining the uniqueness of the authors miR-146-3p finding. Interestingly neither miRNA was significantly differentially expressed in our cell line data, and thus was not investigated in the current human CaP cohort.

This is neither surprising nor uncommon. For example, diagnostic gene expression tests that predict breast cancer progression risks include different gene classifiers but are equally effective and clinically approved [85, 86]. These considerations aside, this recent study [13] combined with the data in the current study provide evidence that indeed miRNA expression levels in serum can be found to associate with biochemical progression and that this association reflects critical events in the tumor.

There is intense interest in the exploitation of serum miRNA patterns as prognostic markers to predict progression risk directly. Uniquely, the present study revealed that miRNA expression patterns in serum, collected before surgery, and combined with PSA are better able to predict early progression risk than merely clinical variables alone (Table 3). That is, the current study remains (to the best of our knowledge) the first to test miRNA prognostic capacity using a cohort of matched patient serum samples. In this capacity we propose that the current study will encourage the development of other prognostic miRNA signatures, either in the same or in different prostate cancer disease settings. The generalizable nature of individual miRNA to predict risk will only emerge from the meta-analyses of such studies.

In turn, these findings have significant potential to reduce the impact of co-morbidities associated with surgery [87, 88] and to identify patients for whom aggressive therapies are warranted. The prospect of being able to exploit serum miRNA expression, as a PCR based assay, either alone or combined with other serum markers (e.g. PSA) has a profound capacity to improve the clinical management of prostate cancer by widespread application of a cost-effective, robust and non-invasive serum measurement.

## 4 MATERIALS AND METHODS

### 4.1 Biological analyses

#### 4.1.1 Cell Lines

Prostate cell lines included those derived from European American men; normal prostate epithelial cells (RWPE-1, HPr1, HPr1AR and PNT2), early stage CaP cells (RWPE-2), androgen sensitive CaP (LNCaP), Androgen deprivation therapy (ADT) recurrent CaP (ADT-RCaP) (LNCaP-C4-2), metastatic CaP (DU145, PC3 and PC3M) and CaP xenograft cell lines (22Rv1 and LAPC4). Cell lines from African American men; clinically localized CaP (E006AA)[23] and ADT recurrent CaP (MDA PCa 2b) [24]. All cells were authenticated at Roswell Park Cancer Institute (RPCI) immediately prior to the start of the study.

2.1.2. RPCI Patient Samples: All serum samples were collected with IRB approval at RPCI. Patient serum and demographic, epidemiologic, clinical and pathology data were collected through the Data Bank and BioRepository (DBBR), a shared resource at RPCI. All blood specimens were collected from newly diagnosed men with CaP, prior to any treatment, and were immediately processed and stored in liquid nitrogen within one hour of blood draw [25]. All men had newly diagnosed clinically localized CaP and had no prior history of either another cancer or other treatment for CaP at the time of blood collection. Standard prognostic variables included clinical (cTNM) and pathological (pTNM) stage, Gleason score, and PSA. All patients had at least 3 years of follow-up data. Biochemical progression [5] was defined by serum PSA of 0.2 ng/mL or greater (obtained 6 weeks – 3 months postoperatively), with a second confirmatory level of PSA greater than 0.2 ng/mL (N=33, classified as progressors), all other men were classified as non-progressors (N=66). The progressors and non-progressors were selected from a larger group of CaP patients who attended urology clinics at RPCI, and for whom there was detailed follow-up (N>450) extending over an 8 year period. From these samples the test cohort was selected by being frequency matched on age and Gleason grade sum using a bootstrapping procedure. For 10 of these selected serum samples, matched tumor samples were also available.

#### 4.1.3 Microarray and Q-RT-PCR analysis

RNA was isolated from cell lines and human tumor samples using Trizol and from serum samples using Qiagen miRNeasy kit with Exiqon recommended modifications. Microarray analysis was performed with Exiqon miRNA 5th generation expression arrays using single color hybridization in biological triplicates for each cell line. Q-RT-PCR analysis was performed using Exiqon miRNA specific primers and reagents in 384 well plates as recommended by Exiqon. All miRNAs with missing values (ct>38) were removed. Samples were then normalized using the overall mean miRNA expression value [26].

### 4.2 Statistical analyses

#### 4.2.1 Microarray

Normalized expression of miRNAs was compared across pairs of cell lines using linear mixed effects models as implemented in LIMMA[27]. Differentially expressed miRNAs were identified using p-value ≤0.05 and fold change ≥1.5 cut-off.

#### 4.2.2 Q-RT-PCR

Differentially expressed miRNAs compared to RWPE-1 were normalized using 5S rRNA (Exiqon reference gene panel) and measured with two-tailed t-test (p-value ≤0.05 and fold change ≥1.5 cut-off). Hierarchical clustering was used to assess if selected miRNAs could be used to differentiate normal epithelial, CaP progression and metastatic cell lines.

#### 4.2.3 *In silico* validation

Publically available data was used to help determine if miRNA expression identified from comparing pairs of cell lines (RWPE-1 vs RWPE-2; HPr1-AR vs LNCaP) correlated with miRNA expression between tumor and normal samples in a subcohort of localized tumors from the Memorial Sloan-Kettering Cancer Center (MSKCC) archive [28]. Specifically, 78 self-reporting White non-Hispanic men with confirmed locally-confined CaP tumors (with 22 corresponding matched normal tissue) obtained from radical prostatectomy were selected for further analyses [28]. Information is publically available on a total of 368 miRNAs of which 367 were common between the microarrays analyzed at RPCI and MSKCC.

#### 4.2.4 Network analyses

MiRNA-targeted networks were identified by pathway enrichment analyses using Diana-mirPath[29].

#### 4.2.5 miRNA serum tumor correlation

Q-RT-PCR was undertaken in 10 CaP matched serum-tumor pairs and Pearson's correlation calculated.

#### 4.2.6 RPCI Patient Samples

The distribution of clinicopathological characteristics was compared among the groups using analysis of variance (ANOVA) for continuous variables and the デ2-test for categorical variables. Continuous variables tested included age at diagnosis, preoperative PSA and body mass index (BMI); Gleason grade sum (greater than 7 vs less than 7) was examined as a categorical variable.

Multivariate Cox proportional hazard models were used to determine the miRNA, pathological and clinical features that were significantly associated with hazard of biochemical progression [30].

Penalized regression models (Lasso models using the R package glmnet [31, 32]) were used to determine what combinations of all measured miRNA, clinical and pathological variables best predicted biochemical progression. These models were also used to determine if either individual or joint combinations of miRNA were at least equivalent and/or better predictors of biochemical progression than clinical variables alone. Specifically, an unrestricted model allowing any parameter to enter/exit the model was compared to a restricted model that forced PSA, Gleason grade sum, and age to remain in the model. The lambda parameter for each model was estimated using 10-fold cross-validation and binomial deviance distance measure.

## Supplementary Figures And Tables





## References

[1] Schroder FH, Hugosson J, Roobol MJ, Tammela TL, Ciatto S, Nelen V, Kwiatkowski M, Lujan M, Lilja H, Zappa M, Denis LJ, Recker F, Berenguer A, Maattanen L, Bangma CH, Aus G (2009). Screening and prostate-cancer mortality in a randomized European study. N Engl J Med.

[2] Andriole GL, Crawford ED, Grubb RL, Buys SS, Chia D, Church TR, Fouad MN, Gelmann EP, Kvale PA, Reding DJ, Weissfeld JL, Yokochi LA, O'Brien B, Clapp JD, Rathmell JM, Riley TL (2009). Mortality results from a randomized prostate-cancer screening trial. N Engl J Med.

[3] Kuriyama M, Wang MC, Papsidero LD, Killian CS, Shimano T, Valenzuela L, Nishiura T, Murphy GP, Chu TM (1980). Quantitation of prostate-specific antigen in serum by a sensitive enzyme immunoassay. Cancer research.

[4] Wang MC, Valenzuela LA, Murphy GP, Chu TM (1979). Purification of a human prostate specific antigen. Investigative urology.

[5] Cookson MS, Aus G, Burnett AL, Canby-Hagino ED, D'Amico AV, Dmochowski RR, Eton DT, Forman JD, Goldenberg SL, Hernandez J, Higano CS, Kraus SR, Moul JW, Tangen C, Thrasher JB, Thompson I (2007). Variation in the definition of biochemical recurrence in patients treated for localized prostate cancer: the American Urological Association Prostate Guidelines for Localized Prostate Cancer Update Panel report and recommendations for a standard in the reporting of surgical outcomes. The Journal of urology.

[6] Freedland SJ (2011). Screening, risk assessment, and the approach to therapy in patients with prostate cancer. Cancer.

[7] Buyyounouski MK, Pickles T, Kestin LL, Allison R, Williams SG (2012). Validating the interval to biochemical failure for the identification of potentially lethal prostate cancer. Journal of clinical oncology : official journal of the American Society of Clinical Oncology.

[8] Watahiki A, Macfarlane RJ, Gleave ME, Crea F, Wang Y, Helgason CD, Chi KN (2013). Plasma miRNAs as Biomarkers to Identify Patients with Castration-Resistant Metastatic Prostate Cancer. International journal of molecular sciences.

[9] Tsuchiyama K, Ito H, Taga M, Naganuma S, Oshinoya Y, Nagano K, Yokoyama O, Itoh H (2013). Expression of MicroRNAs associated with Gleason grading system in prostate cancer: miR-182-5p is a useful marker for high grade prostate cancer. The Prostate.

[10] Sun D, Layer R, Mueller AC, Cichewicz MA, Negishi M, Paschal BM, Dutta A (2013). Regulation of several androgen-induced genes through the repression of the miR-99a/let-7c/miR-125b-2 miRNA cluster in prostate cancer cells. Oncogene.

[11] Sita-Lumsden A, Dart DA, Waxman J, Bevan CL (2013). Circulating microRNAs as potential new biomarkers for prostate cancer. British journal of cancer.

[12] Selth LA, Townley SL, Gillis JL, Tilley WD, Butler LM (2013). Identification of Prostate Cancer-Associated MicroRNAs in Circulation Using a Mouse Model of Disease. Methods in molecular biology.

[13] Selth LA, Townley SL, Bert AG, Stricker PD, Sutherland PD, Horvath LG, Goodall GJ, Butler LM, Tilley WD (2013). Circulating microRNAs predict biochemical recurrence in prostate cancer patients. British journal of cancer.

[14] Wang L, Tang H, Thayanithy V, Subramanian S, Oberg AL, Cunningham JM, Cerhan JR, Steer CJ, Thibodeau SN (2009). Gene networks and microRNAs implicated in aggressive prostate cancer. Cancer research.

[15] Tong AW, Fulgham P, Jay C, Chen P, Khalil I, Liu S, Senzer N, Eklund AC, Han J, Nemunaitis J (2009). MicroRNA profile analysis of human prostate cancers. Cancer gene therapy.

[16] Siva AC, Nelson LJ, Fleischer CL, Majlessi M, Becker MM, Vessella RL, Reynolds MA (2009). Molecular assays for the detection of microRNAs in prostate cancer. Molecular cancer.

[17] Mitchell PS, Parkin RK, Kroh EM, Fritz BR, Wyman SK, Pogosova-Agadjanyan EL, Peterson A, Noteboom J, O'Briant KC, Allen A, Lin DW, Urban N, Drescher CW, Knudsen BS, Stirewalt DL, Gentleman R (2008). Circulating microRNAs as stable blood-based markers for cancer detection. Proceedings of the National Academy of Sciences of the United States of America.

[18] Ambs S, Prueitt RL, Yi M, Hudson RS, Howe TM, Petrocca F, Wallace TA, Liu CG, Volinia S, Calin GA, Yfantis HG, Stephens RM, Croce CM (2008). Genomic profiling of microRNA and messenger RNA reveals deregulated microRNA expression in prostate cancer. Cancer research.

[19] Lussier YA, Stadler WM, Chen JL (2012). Advantages of genomic complexity: bioinformatics opportunities in microRNA cancer signatures. Journal of the American Medical Informatics Association : JAMIA.

[20] Nguyen HC, Xie W, Yang M, Hsieh CL, Drouin S, Lee GS, Kantoff PW (2012). Expression differences of circulating microRNAs in metastatic castration resistant prostate cancer and low-risk, localized prostate cancer. Prostate.

[21] Mitchell PS, Parkin RK, Kroh EM, Fritz BR, Wyman SK, Pogosova-Agadjanyan EL, Peterson A, Noteboom J, O'Briant KC, Allen A, Lin DW, Urban N, Drescher CW, Knudsen BS, Stirewalt DL, Gentleman R (2008). Circulating microRNAs as stable blood-based markers for cancer detection. Proc Natl Acad Sci U S A.

[22] Selth LA, Tilley WD, Butler LM (2012). Circulating microRNAs - macro-utility as markers of prostate cancer?. Endocr Relat Cancer.

[23] Koochekpour S, Maresh GA, Katner A, Parker-Johnson K, Lee TJ, Hebert FE, Kao YS, Skinner J, Rayford W (2004). Establishment and characterization of a primary androgen-responsive African-American prostate cancer cell line, E006AA. Prostate.

[24] Navone NM, Olive M, Ozen M, Davis R, Troncoso P, Tu SM, Johnston D, Pollack A, Pathak S, von Eschenbach AC, Logothetis CJ (1997). Establishment of two human prostate cancer cell lines derived from a single bone metastasis. Clin Cancer Res.

[25] Ambrosone CB (2006). Sample collection, processing, and storage for large-scale studies: biorepositories to support cancer research. Cancer epidemiology, biomarkers & prevention : a publication of the American Association for Cancer Research, cosponsored by the American Society of Preventive Oncology.

[26] Mestdagh P, Van Vlierberghe P, De Weer A, Muth D, Westermann F, Speleman F, Vandesompele J (2009). A novel and universal method for microRNA RT-qPCR data normalization. Genome Biol.

[27] Smyth GK (2004). Linear models and empirical Bayes methods for assessing differential expression in microarray experiments. Stat Appl Genet Mol Biol.

[28] Taylor BS, Schultz N, Hieronymus H, Gopalan A, Xiao Y, Carver BS, Arora VK, Kaushik P, Cerami E, Reva B, Antipin Y, Mitsiades N, Landers T, Dolgalev I, Major JE, Wilson M (2010). Integrative genomic profiling of human prostate cancer. Cancer Cell.

[29] Papadopoulos GL, Alexiou P, Maragkakis M, Reczko M, Hatzigeorgiou AG (2009). DIANA-mirPath: Integrating human and mouse microRNAs in pathways. Bioinformatics.

[30] Cox DR (1972). Regression Models and Life-Tables. Journal of the Royal Statistical Society.

[31] Friedman J, Hastie T, Tibshirani R (2010). Regularization Paths for Generalized Linear Models via Coordinate Descent. J Stat Softw.

[32] Noah Simon JHF, Trevor Hastie, Rob Tibshirani (2011). Regularization Paths for Cox's Proportional Hazards Model via Coordinate Descent. Journal of Statistical Software.

[33] Bello D, Webber MM, Kleinman HK, Wartinger DD, Rhim JS (1997). Androgen responsive adult human prostatic epithelial cell lines immortalized by human papillomavirus 18. Carcinogenesis.

[34] Ling MT, Chan KW, Choo CK (2001). Androgen induces differentiation of a human papillomavirus 16 E6/E7 immortalized prostate epithelial cell line. J Endocrinol.

[35] Thalmann GN, Anezinis PE, Chang SM, Zhau HE, Kim EE, Hopwood VL, Pathak S, von Eschenbach AC, Chung LW (1994). Androgen-independent cancer progression and bone metastasis in the LNCaP model of human prostate cancer. Cancer Res.

[36] Watahiki A, Wang Y, Morris J, Dennis K, O'Dwyer HM, Gleave M, Gout PW (2011). MicroRNAs associated with metastatic prostate cancer. PLoS ONE.

[37] Leite KR, Tomiyama A, Reis ST, Sousa-Canavez JM, Sanudo A, Camara-Lopes LH, Srougi M (2011). MicroRNA expression profiles in the progression of prostate cancer-from high-grade prostate intraepithelial neoplasia to metastasis. Urol Oncol.

[38] Martens-Uzunova ES, Jalava SE, Dits NF, van Leenders GJ, Moller S, Trapman J, Bangma CH, Litman T, Visakorpi T, Jenster G (2012). Diagnostic and prognostic signatures from the small non-coding RNA transcriptome in prostate cancer. Oncogene.

[39] Schaefer A, Jung M, Mollenkopf HJ, Wagner I, Stephan C, Jentzmik F, Miller K, Lein M, Kristiansen G, Jung K (2010). Diagnostic and prognostic implications of microRNA profiling in prostate carcinoma. Int J Cancer.

[40] Volinia S, Calin GA, Liu CG, Ambs S, Cimmino A, Petrocca F, Visone R, Iorio M, Roldo C, Ferracin M, Prueitt RL, Yanaihara N, Lanza G, Scarpa A, Vecchione A, Negrini M (2006). A microRNA expression signature of human solid tumors defines cancer gene targets. Proc Natl Acad Sci U S A.

[41] Ozen M, Creighton CJ, Ozdemir M, Ittmann M (2008). Widespread deregulation of microRNA expression in human prostate cancer. Oncogene.

[42] Porkka KP, Pfeiffer MJ, Waltering KK, Vessella RL, Tammela TL, Visakorpi T (2007). MicroRNA expression profiling in prostate cancer. Cancer Res.

[43] Brase JC, Johannes M, Schlomm T, Falth M, Haese A, Steuber T, Beissbarth T, Kuner R, Sultmann H (2011). Circulating miRNAs are correlated with tumor progression in prostate cancer. International journal of cancer Journal international du cancer.

[44] Dai Y, Xie CH, Neis JP, Fan CY, Vural E, Spring PM (2011). MicroRNA expression profiles of head and neck squamous cell carcinoma with docetaxel-induced multidrug resistance. Head Neck.

[45] Leskela S, Leandro-Garcia LJ, Mendiola M, Barriuso J, Inglada-Perez L, Munoz I, Martinez-Delgado B, Redondo A, de Santiago J, Robledo M, Hardisson D, Rodriguez-Antona C (2011). The miR-200 family controls beta-tubulin III expression and is associated with paclitaxel-based treatment response and progression-free survival in ovarian cancer patients. Endocr Relat Cancer.

[46] Bhatnagar N, Li X, Padi SK, Zhang Q, Tang MS, Guo B (2010). Downregulation of miR-205 and miR-31 confers resistance to chemotherapy-induced apoptosis in prostate cancer cells. Cell Death Dis.

[47] Creighton CJ, Fountain MD, Yu Z, Nagaraja AK, Zhu H, Khan M, Olokpa E, Zariff A, Gunaratne PH, Matzuk MM, Anderson ML (2010). Molecular profiling uncovers a p53-associated role for microRNA-31 in inhibiting the proliferation of serous ovarian carcinomas and other cancers. Cancer Res.

[48] Valastyan S, Chang A, Benaich N, Reinhardt F, Weinberg RA (2010). Concurrent suppression of integrin alpha5, radixin, and RhoA phenocopies the effects of miR-31 on metastasis. Cancer Res.

[49] Bello-DeOcampo D, Tindall DJ (2003). TGF-betal/Smad signaling in prostate cancer. Curr Drug Targets.

[50] Huang K, Zhang JX, Han L, You YP, Jiang T, Pu PY, Kang CS MicroRNA roles in beta-catenin pathway. Molecular cancer.

[51] Yang Y, Ahn YH, Gibbons DL, Zang Y, Lin W, Thilaganathan N, Alvarez CA, Moreira DC, Creighton CJ, Gregory PA, Goodall GJ, Kurie JM (2011). The Notch ligand Jagged2 promotes lung adenocarcinoma metastasis through a miR-200-dependent pathway in mice. The Journal of clinical investigation.

[52] Gregory PA, Bert AG, Paterson EL, Barry SC, Tsykin A, Farshid G, Vadas MA, Khew-Goodall Y, Goodall GJ (2008). The miR-200 family and miR-205 regulate epithelial to mesenchymal transition by targeting ZEB1 and SIP1. Nature cell biology.

[53] Ding Z, Wu CJ, Chu GC, Xiao Y, Ho D, Zhang J, Perry SR, Labrot ES, Wu X, Lis R, Hoshida Y, Hiller D, Hu B, Jiang S, Zheng H, Stegh AH (2011). SMAD4-dependent barrier constrains prostate cancer growth and metastatic progression. Nature.

[54] Thorne JL, Maguire O, Doig CL, Battaglia S, Fehr L, Sucheston LE, Heinaniemi M, O'Neill LP, McCabe CJ, Turner BM, Carlberg C, Campbell MJ (2011). Epigenetic control of a VDR-governed feed-forward loop that regulates p21(waf1/cip1) expression and function in non-malignant prostate cells. Nucleic Acids Res.

[55] Singh PK, Campbell MJ (2013). The interaction of microRNA and epigenetic modifications in prostate cancer. Cancers.

[56] Medina R, Zaidi SK, Liu CG, Stein JL, van Wijnen AJ, Croce CM, Stein GS (2008). MicroRNAs 221 and 222 bypass quiescence and compromise cell survival. Cancer research.

[57] Mercatelli N, Coppola V, Bonci D, Miele F, Costantini A, Guadagnoli M, Bonanno E, Muto G, Frajese GV, De Maria R, Spagnoli LG, Farace MG, Ciafre SA (2008). The inhibition of the highly expressed miR-221 and miR-222 impairs the growth of prostate carcinoma xenografts in mice. PLoS One.

[58] Lin D, Cui F, Bu Q, Yan C (2011). The expression and clinical significance of GTP-binding RAS-like 3 (ARHI) and microRNA 221 and 222 in prostate cancer. J Int Med Res.

[59] Tong AW, Fulgham P, Jay C, Chen P, Khalil I, Liu S, Senzer N, Eklund AC, Han J, Nemunaitis J (2009). MicroRNA profile analysis of human prostate cancers. Cancer Gene Ther.

[60] Sun T, Wang Q, Balk S, Brown M, Lee GS, Kantoff P (2009). The role of microRNA-221 and microRNA-222 in androgen-independent prostate cancer cell lines. Cancer Res.

[61] Wach S, Nolte E, Szczyrba J, Stohr R, Hartmann A, Orntoft T, Dyrskjot L, Eltze E, Wieland W, Keck B, Ekici AB, Grasser F, Wullich B (2012). MicroRNA profiles of prostate carcinoma detected by multiplatform microRNA screening. International journal of cancer Journal international du cancer.

[62] Lee YS, Kim HK, Chung S, Kim KS, Dutta A (2005). Depletion of human micro-RNA miR-125b reveals that it is critical for the proliferation of differentiated cells but not for the down-regulation of putative targets during differentiation. J Biol Chem.

[63] Yu D, Zhou H, Xun Q, Xu X, Ling J, Hu Y (2012). microRNA-103 regulates the growth and invasion of endometrial cancer cells through the downregulation of tissue inhibitor of metalloproteinase 3. Oncol Lett.

[64] Gottardo F, Liu CG, Ferracin M, Calin GA, Fassan M, Bassi P, Sevignani C, Byrne D, Negrini M, Pagano F, Gomella LG, Croce CM, Baffa R (2007). Micro-RNA profiling in kidney and bladder cancers. Urol Oncol.

[65] Martello G, Rosato A, Ferrari F, Manfrin A, Cordenonsi M, Dupont S, Enzo E, Guzzardo V, Rondina M, Spruce T, Parenti AR, Daidone MG, Bicciato S, Piccolo S (2010). A MicroRNA targeting dicer for metastasis control. Cell.

[66] Felli N, Fontana L, Pelosi E, Botta R, Bonci D, Facchiano F, Liuzzi F, Lulli V, Morsilli O, Santoro S, Valtieri M, Calin GA, Liu CG, Sorrentino A, Croce CM, Peschle C (2005). MicroRNAs 221 and 222 inhibit normal erythropoiesis and erythroleukemic cell growth via kit receptor down-modulation. Proc Natl Acad Sci U S A.

[67] Ciafre SA, Galardi S, Mangiola A, Ferracin M, Liu CG, Sabatino G, Negrini M, Maira G, Croce CM, Farace MG (2005). Extensive modulation of a set of microRNAs in primary glioblastoma. Biochem Biophys Res Commun.

[68] Hao J, Zhang C, Zhang A, Wang K, Jia Z, Wang G, Han L, Kang C, Pu P (2012). miR-221/222 is the regulator of Cx43 expression in human glioblastoma cells. Oncol Rep.

[69] Nikiforova MN, Tseng GC, Steward D, Diorio D, Nikiforov YE (2008). MicroRNA expression profiling of thyroid tumors: biological significance and diagnostic utility. J Clin Endocrinol Metab.

[70] Keutgen XM, Filicori F, Crowley MJ, Wang Y, Scognamiglio T, Hoda R, Buitrago D, Cooper D, Zeiger MA, Zarnegar R, Elemento O, Fahey TJ (2012). A panel of four miRNAs accurately differentiates malignant from benign indeterminate thyroid lesions on fine needle aspiration. Clin Cancer Res.

[71] Li N, Tang B, Zhu ED, Li BS, Zhuang Y, Yu S, Lu DS, Zou QM, Xiao B, Mao XH (2012). Increased miR-222 in H. pylori-associated gastric cancer correlated with tumor progression by promoting cancer cell proliferation and targeting RECK. FEBS Lett.

[72] Wang J, Wang Q, Liu H, Hu B, Zhou W, Cheng Y (2010). MicroRNA expression and its implication for the diagnosis and therapeutic strategies of gastric cancer. Cancer Lett.

[73] Kim BH, Hong SW, Kim A, Choi SH, Yoon SO (2012). Prognostic implications for high expression of oncogenic microRNAs in advanced gastric carcinoma. J Surg Oncol.

[74] Papaconstantinou IG, Manta A, Gazouli M, Lyberopoulou A, Lykoudis PM, Polymeneas G, Voros D (2012). Expression of MicroRNAs in Patients With Pancreatic Cancer and Its Prognostic Significance. Pancreas.

[75] Puerta-Gil P, Garcia-Baquero R, Jia AY, Ocana S, Alvarez-Mugica M, Alvarez-Ossorio JL, Cordon-Cardo C, Cava F, Sanchez-Carbayo M (2012). miR-143, miR-222, and miR-452 are useful as tumor stratification and noninvasive diagnostic biomarkers for bladder cancer. Am J Pathol.

[76] Zhao JJ, Lin J, Yang H, Kong W, He L, Ma X, Coppola D, Cheng JQ (2008). MicroRNA-221/222 negatively regulates estrogen receptor alpha and is associated with tamoxifen resistance in breast cancer. J Biol Chem.

[77] Miller TE, Ghoshal K, Ramaswamy B, Roy S, Datta J, Shapiro CL, Jacob S, Majumder S (2008). MicroRNA-221/222 confers tamoxifen resistance in breast cancer by targeting p27Kip1. J Biol Chem.

[78] Wach S, Nolte E, Szczyrba J, Stohr R, Hartmann A, Orntoft T, Dyrskjot L, Eltze E, Wieland W, Keck B, Ekici AB, Grasser F, Wullich B (2012). MicroRNA profiles of prostate carcinoma detected by multiplatform microRNA screening. International journal of cancer Journal international du cancer.

[79] Cortez MA, Bueso-Ramos C, Ferdin J, Lopez-Berestein G, Sood AK, Calin GA (2011). MicroRNAs in body fluids-the mix of hormones and biomarkers. Nature reviews Clinical oncology.

[80] Shi XB, Xue L, Yang J, Ma AH, Zhao J, Xu M, Tepper CG, Evans CP, Kung HJ, deVere White RW (2007). An androgen-regulated miRNA suppresses Bak1 expression and induces androgen-independent growth of prostate cancer cells. Proc Natl Acad Sci U S A.

[81] Yang X, Bemis L, Su LJ, Gao D, Flaig TW (2012). miR-125b Regulation of Androgen Receptor Signaling Via Modulation of the Receptor Complex Co-Repressor NCOR2. BioResearch open access.

[82] Di Vizio D, Morello M, Dudley AC, Schow PW, Adam RM, Morley S, Mulholland D, Rotinen M, Hager MH, Insabato L, Moses MA, Demichelis F, Lisanti MP, Wu H, Klagsbrun M, Bhowmick NA (2012). Large oncosomes in human prostate cancer tissues and in the circulation of mice with metastatic disease. Am J Pathol.

[83] Palma J, Yaddanapudi SC, Pigati L, Havens MA, Jeong S, Weiner GA, Weimer KM, Stern B, Hastings ML, Duelli DM (2012). MicroRNAs are exported from malignant cells in customized particles. Nucleic acids research.

[84] Pigati L, Yaddanapudi SC, Iyengar R, Kim DJ, Hearn SA, Danforth D, Hastings ML, Duelli DM (2010). Selective release of microRNA species from normal and malignant mammary epithelial cells. PLoS One.

[85] Ignatiadis M, Desmedt C (2007). Predicting risk of breast cancer recurrence using gene-expression profiling. Pharmacogenomics.

[86] Li Q, Seo JH, Stranger B, McKenna A, Pe'er I, Laframboise T, Brown M, Tyekucheva S, Freedman ML (2013). Integrative eQTL-based analyses reveal the biology of breast cancer risk loci. Cell.

[87] Batura D, and Gopal, Rao G (2012). The national burden of infections after prostate biopsy in England and Wales: a wake-up call for better prevention. J Antimicrob Chemother.

[88] Glaser AP, Novakovic K, Helfand BT (2012). The Impact of Prostate Biopsy on Urinary Symptoms, Erectile Function, and Anxiety. Curr Urol Rep.

