# Supplementary material for: Serum microRNA expression patterns that predict early treatment failure in prostate cancer patients
Source: Oncotarget. 2014 Feb 13;5(3):824–40. doi: 10.18632/oncotarget.1776 (PMC3996656; doi:10.18632/oncotarget.1776)
Supplement: Supplementary file 1 [file oncotarget-05-824-s001.pdf]

Serum microRNA expression patterns that predict early treatment failure in prostate cancer patients – Singh et al

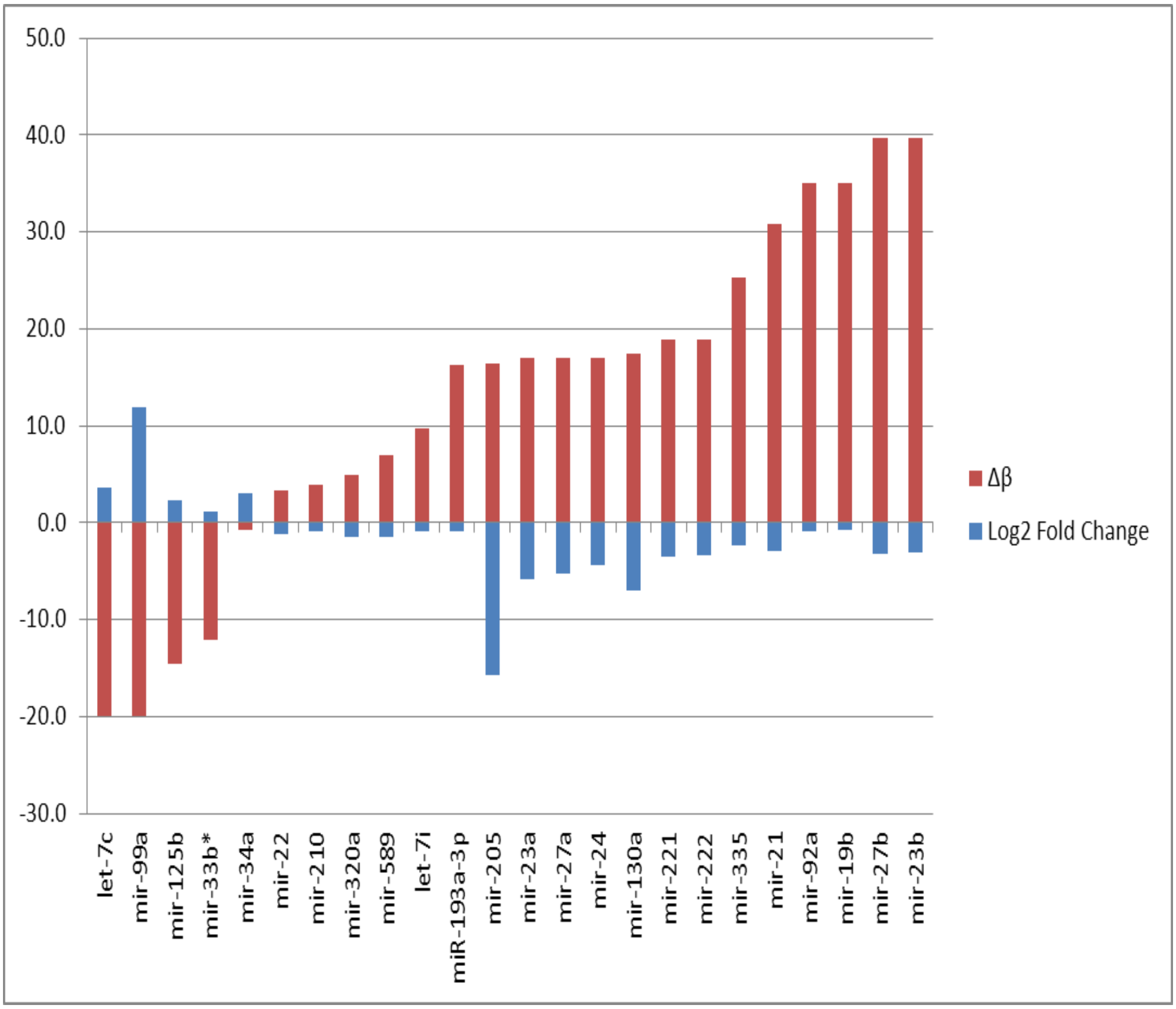

Supplementary Figure 1. Correlation of miRNA expression with CpG methylation status in LNCaP cells. Log2 miRNA expression in LNCaP cells compared to non-malignant RWPE1 cells is shown by blue line. For CpG methylation status comparison, methylation data for LNCaP and non-malignant PrEC cells was downloaded from ENCODE. CpG in ± 5kb flanking region around miRNAs genomic locations were considered as associative with that specific miRNA. The mean of beta values for all the CpGs in ± 5kb region was calculated for both Prec and LNCaP. The delta beta valued were calculated as Δβ= Meanβ, LNCaP- Meanβ, Prec. Delta beta valued with negative values shows lower methylation in LNCaP and postive delta beta values shows higher methylation in LNCaP compared to PrEC cells.

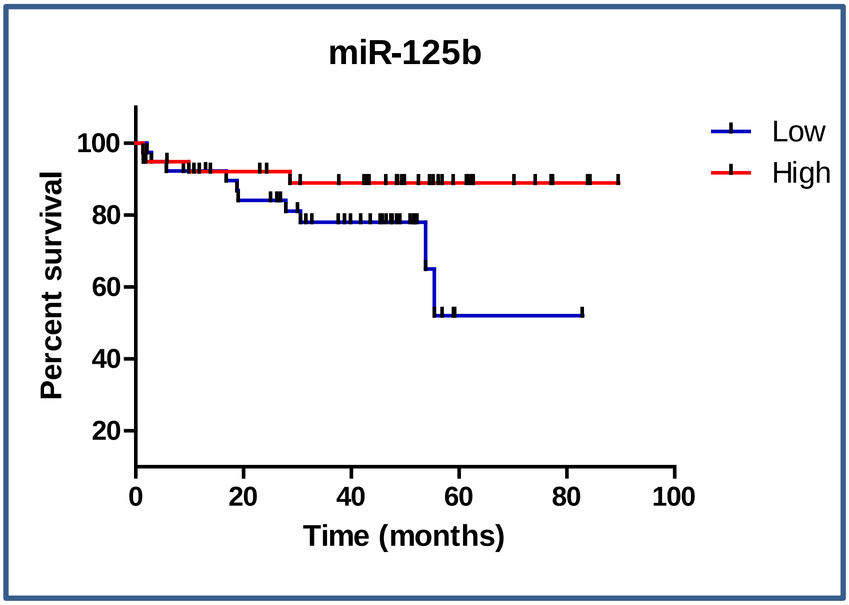

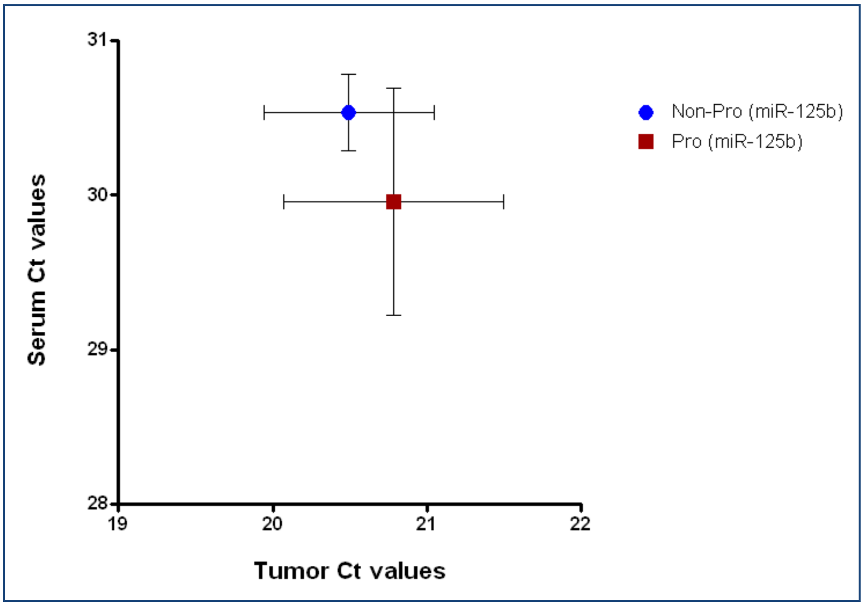

Supplementary Figure 2. MiR-125b tumor expression predicts biochemical progression in the MSKCC primary CaP patient cohort. Top. MiRNA expression and clinical data was downloaded and analyzed from the cbio genomics portal (http://www.cbioportal.org/public-portal/). 78 white, non-hispanic patients were available and were considered in the analysis. For each miRNA (miR-222, miR-103, miR-125b), patients were separated into low and high expression using the median expression value as a threshold. Survival analysis (time to biochemical recurrence) was undertaken using GraphPad Prism 5 software. Bottom. Expression of miR-125b was measured in the tumor and serum. Bottom. miR-125b was measured in 10 patients for whom matched tumor and serum was available by Q-RT-PCR. The patients were 6 men who did not progress (Non-Pro), and 4 men who did progress (Pro). The Ct values are indicated in the tumor and serum for each patient. The progressors displayed lower levels (higher Ct values) of miR-125b in the tumor and higher levels (lower Ct values) in the serum compared to the non-progressors.
