# Supplementary material for: Serum microRNA expression patterns that predict early treatment failure in prostate cancer patients
Source: Oncotarget. 2014 Feb 13;5(3):824–40. doi: 10.18632/oncotarget.1776 (PMC3996656; doi:10.18632/oncotarget.1776)
Supplement: Supplementary file 2 [file oncotarget-05-824-s002.pdf]

Serum microRNA expression patterns that predict early treatment failure in prostate cancer patients – Singh et al

**miRNA**	**Normalized Mean CT (before therapy)**	**Normalized Mean CT (after therapy)**	**Log2 Fold Change**	**P Value**
hsa-miR-320a	2.931	1.991	-0.940	0.0001
hsa-let-7b	3.157	1.798	-1.360	0.0003
hsa-let-7c	-3.492	-4.488	-0.995	0.0004
hsa-miR-92a	3.881	2.995	-0.885	0.0038
hsa-miR-130a	-2.425	-1.793	0.633	0.0050
hsa-miR-21	1.804	2.400	0.595	0.0099
hsa-miR-29b	-5.283	-4.584	0.700	0.0106
hsa-miR-27a	0.509	1.277	0.768	0.0136
hsa-miR-19b	2.449	2.927	0.478	0.0194
hsa-miR-222	-0.110	-0.480	-0.371	0.0424
hsa-miR-335	-3.219	-2.636	0.583	0.0449

Supplementary Table 1: miRNA expression patterns expression after 3 months of androgen deprivation therapy. A cohort of men with hormone sensitive prostate cancer (HSPC, N=17) with treated with anti-androgen therapy and expression of 34 miRNA (see Table 1) were measured in serum sample collected before and three months after treatment. 11 miRNA differed between time points, the most significant, miR-320a (log_2 fold change =-0.92, p<.0001. Of the miRNAs identified as differentially expressed in the men with localized disease, miR-222 was also differentially expressed between the time points (log_2 fold change=-0.37, p<.04).

PMID	Author	Year	Study design	Sample	miR exp Method	Normalization	Results
23935962	Cheng HH	2013	25 mCRPC vs 25 age-matched controls, replication set 21 mCRPC patients and 20 age-matched healthy controls	Serum	365 miRs, TaqMan Low-Density Arrays (TLDA)	spike-in control miRNAs	miR-141, miR-200a, miR-200c, miR-375 and miR-210
23846169	Selth LA	2013	BCR (n=8) or no BCR (n=8), validation set BCR(n=31) no BCR (n=39)	Serum	TaqMan Low-Density Arrays (TLDA), MicroRNA A&B Cards Set v3.0	global normalisation	miR-194
22887127	Nguyen HC	2013	28 localized disease vs 30 of high-risk localized disease, 26 of metastatic CRPC with ADT	serum	669 miRs, TaqMan miR Arrays	U6-small nuclear RNA	miR-375, miR-378*, miR-141, miR-409-3p
22298119	Shen J	2012	82 PCa patients, comparison between different groups based on CAPRA Score and D'Amico Score	Plasma	4 miRs analysed, TaqMan, absolute copy numbers		Four miRNAs (miR-20a, miR-21, miR-145, and miR-221) could also distinguish high versus low risk in PCa patients by D'Amico score with an AUC of 0.824.
22298030	Chen ZH,	2012	25 CaP (15 nonmetastatic patients, 10 metastatic patients), 17 BPH (training set), 80 CaP, 44 BPH, and 54 healthy controls (validation set)	Plasma	Illumina's miRNA expression for Training	U6	3 down (let-7e, let-7c, miR-30c) and 4 upregulated (miR-622, miR-1285). 5 miRNAs could differentiate CaP from BPH and healthy controls with high diagnosis performance, with an AUC of 0.924 and 0.860, respectively.
22240788	Bryant RJ	2012	78 CaP and 28 normal control	plasma	qRT-PCR (742 miRNAs)	Cel-miR-39	12 miRNAs altered in PCa patients compared with healthy controls. 16 miRNAs altered in metastatic vs localized PCa (including miR-141 and miR-375)

22052531	Selth LA	2012	14 TRAMP mice, 14 healthy controls. Validation: 25 patients (metastatic CRPC), 25 healthy controls	Serum	Microarray (Affymetrix), qRT-PCR (609 murine miRNAs, 10 human miRNAs)	cel-miR-39	miR-141, miR-298, miR-346, and miR-375 levels elevated in patients
21487968	Zheng C	2012	normal persons (n = 20), androgen-dependent prostate (n = 15), and androgen-independent prostate (n = 8)	plasma	miR-221	RNU6B	incresed miR-221 expression in the ADPC and AIPC patientscompared to normal donors, while miR-221 expression was up-regulated in ADPC patients compared with AIPC patients
21539977	Mahn R	2011	45 patients (37 localized PCa, 8 metastatic), 18 BPH controls, 20 healthy controls	Serum	qRT-PCR (5 miRNAs)	miR-16 , RNU1A	miR-26a, miR-195, and let-7i levels elevated in PCa compared with BPH samples
20473869	Brase JC	2011	7 high-grade, 14 low-grade patients. Validation: 116 patients (various grades)	serum	667 miRNAs, low-density Taqman arrays	Cel spikein miR	miR-141, miR-200b, and miR-375 were elevated in serum from high-grade patients and correlated with clinicopathological parameters
21723797	Gonzales JC	2011	21 CaP patients to the levels of prostate specific antigen (PSA), circulating tumor cells (CTC) and lactate dehydrogenase (LDH)	plasma	miR-141		miR-141 levels predicted clinical outcomes with an odds ratio of at least 8.3
21098088	Moltzahn F	2011	12 healthy male, 36 CaP, 12 low-risk (CAPRA score 1), 12 intermediate-risk (CAPRA score 4), and 12 high-risk [CAPRA score > 5, N+ (regional lymph nodes)]	serum	qRT-PCR (677 miRNAs) TaqMan	median-normalized	Ten miRNAs (miR-20b, -24, -26b, -30c, -93, -106a, -223, -874, -1207-5p, and -1274a) were substantially different between the healthy and all malignant samples.

21274675	Yaman Agaoglu F	2011	51 patients (18 localized PCa, 8 local advanced, 25 metastatic), 20 healthy controls	plasma	qRT-PCR (miR-21, miR-141, and miR-221)	RNU1A	miR-21 and miR-221 levels elevated in PCa patients compared with healthy controls. miR-21, miR-141, and miR-221 levels higher in metastatic vs localized disease
20842666	Zhang HL	2011	56 patients (20 with localized CaP, 20 with androgen-dependent prostate cancer,10 with hormone-refractory prostate cancer (HRPC), and 6 with benign prostatic hyperplasia (BPH))	serum	miR-21	U6 snRNA	miR-21 levels elevated in CRPC patients compared with BPH. miR-21 associated with resistance to docetaxel in CRPC patients
19597549	Lodes MJ	2009	6 patients (stages 2–4 PCa), 8 healthy controls	serum	Microarray (custom) (547 miRNAs)		15 miRNAs were elevated in PCa patients. However, serum miRNAs could not distinguish between different cancer types
18663219	Mitchell PS	2008	25 patients (metastatic PCa), 25 healthy controls	Plasma	qRT-PCR (6 miRNAs) miR-100, miR-125b, miR-141, miR-143, miR-205, and miR-296	Spiked-In C. elegan	miR-141 levels could differentiate PCa patients from healthy subjects

Supplementary Table 2: Key published findings in prostate cancer that have examined the capacity of miRNA to predict tumor progression risk or disease state.

Supplementary Table 3: Pathway enrichment analysis through DIANEpath of miRNA signatures identified in microarray analysis.

Cells in yellow color show pathways common between all three comparisons

RWPE-1 vs RWPE-2			LNCaP vs LNCaP-C4-2			HPr1AR vs LNCaP		
KEGG Pathway	Union	-ln(p-value) (Union)	KEGG Pathway	Union	-ln(p-value) (Union)	KEGG Pathway	Union	-ln(p-value) (Union)
Focal adhesion	71	18.87	Focal adhesion	65	23.34	Wnt signaling pathway	89	25.38
Wnt signaling pathway	57	17.78	MAPK signaling pathway	74	17.87	Axon guidance	78	24.53
Axon guidance	50	17.04	Prostate cancer	35	17.7	Ribosome	1	22.49
TGF-beta signaling pathway	39	15.78	Axon guidance	42	14.99	Focal adhesion	107	22.19
Ubiquitin mediated proteolysis	49	13.66	Insulin signaling pathway	45	14.32	MAPK signaling pathway	131	20.96
Adherens junction	32	13.59	Glioma	26	14.01	Oxidative phosphorylation	10	20.03
MAPK signaling pathway	80	12.69	ErbB signaling pathway	32	13.46	Adherens junction	48	17.23
Oxidative phosphorylation	5	11.07	ECM-receptor interaction	30	13.29	Colorectal cancer	54	16.52
Prostate cancer	35	10.92	Wnt signaling pathway	45	12.35	Regulation of actin cytoskeleton	106	15.5
Chronic myeloid leukemia	31	10.5	Renal cell carcinoma	26	11.76	Renal cell carcinoma	45	15.17
Adipocytokine signaling pathway	29	10.31	mTOR signaling pathway	20	11.59	Melanogenesis	59	14.94
ErbB signaling pathway	34	10.1	Small cell lung cancer	29	10.1	TGF-beta signaling pathway	54	14.15
Regulation of actin cytoskeleton	64	8.98	Oxidative phosphorylation	3	9.91	Ubiquitin mediated proteolysis	71	13.39
GnRH signaling pathway	35	8.94	Chronic myeloid leukemia	26	9.2	ErbB signaling pathway	52	12.96
Glioma	26	8.83	Circadian rhythm	8	9.13	Prostate cancer	52	12.43
Colorectal cancer	32	8.53	TGF-beta signaling pathway	29	8.89	Glioma	40	11.89
Small cell lung cancer	32	8.53	Regulation of actin cytoskeleton	54	8.47	Metabolism of xenobiotics by cytochrome P450	4	11.8
Renal cell carcinoma	27	8.21	Ribosome	1	8.32	Pancreatic cancer	44	11.46
Metabolism of xenobiotics by cytochrome P450	1	7.6	Melanoma	24	7.97	Chronic myeloid leukemia	45	10.98
Pancreatic cancer	27	6.92	T cell receptor signaling pathway	28	7.77	GnRH signaling pathway	52	9.65

Supplementary Table 4: Correlation of miRNA expression in LNCaP cells compared to RWPE1 cells with CpG methylation status around miRNA in LNCaP cells compared to PrEC cells from ENCODE.

Red color cells show high miRNA expression/High methylation in LNCaP cells.
Green color cells show low miRNA expression/low methylation in LNCaP cells.

	Positve results								Correlation between miR expression and methylation
**Chr**	**Start**	**End**	**miR**	**Avg-LNCa**	**Avg-Prec**	**Delta**	**FC LNCaP**	**Delta**	**R2**
chr21	17911066	17912009	hsa-let-7c	621.75	820.50	-198.75	3.612	-19.875	-0.559
chr21	17911066	17912009	hsa-mir-99a	621.75	820.50	-198.75	11.930	-19.875	
chr21	17960584	17962174	hsa-mir-125b	638.00	783.60	-145.60	2.267	-14.560	
chr17	17712609	17721644	hsa-mir-33b*	600.04	720.43	-120.39	1.140	-12.039	
chr1	9210823	9212804	hsa-mir-34a	791.00	798.80	-7.80	3.097	-0.780	
chr17	1613017	1620825	hsa-mir-22	136.00	103.41	32.59	-1.205	3.259	
chr11	563860	569637	hsa-mir-210	307.50	268.58	38.92	-0.917	3.892	
chr8	22102032	22106621	hsa-mir-320a	146.71	96.71	50.00	-1.532	5.000	
chr7	5530946	5540584	hsa-mir-589	621.93	551.81	70.11	-1.432	7.011	
chr12	62992859	63001419	hsa-let-7i	256.67	159.53	97.13	-0.888	9.713	
chr17	29885787	29890860	hsa-miR-193a-3p	485.31	322.85	162.46	-0.945	16.246	
chr1	2.1E+08	2.1E+08	hsa-mir-205	298.93	134.00	164.93	-15.690	16.493	
chr19	13942611	13951895	hsa-mir-23a	610.25	439.80	170.45	-5.845	17.045	
chr19	13942611	13951895	hsa-mir-24	610.25	439.80	170.45	-4.430	17.045	
chr19	13942611	13951895	hsa-mir-27a	610.25	439.80	170.45	-5.292	17.045	
chr11	57405372	57412710	hsa-mir-130a	508.00	334.25	173.75	-6.956	17.375	
chrX	45606471	45607488	hsa-mir-221	868.33	679.33	189.00	-3.560	18.900	
chrX	45606471	45607488	hsa-mir-222	868.33	679.33	189.00	-3.337	18.900	
chr7	1.3E+08	1.3E+08	hsa-mir-335	829.19	576.15	253.04	-2.383	25.304	
chr17	57915665	57918732	hsa-mir-21	430.67	122.92	307.75	-2.900	30.775	
chrX	1.33E+08	1.33E+08	hsa-mir-19b	740.00	390.23	349.77	-0.672	34.977	
chrX	1.33E+08	1.33E+08	hsa-mir-92a	740.00	390.23	349.77	-0.930	34.977	
chr9	97844901	97851424	hsa-mir-23b	806.78	410.00	396.78	-3.113	39.678	
chr9	97844901	97851424	hsa-mir-27b	806.78	410.00	396.78	-3.230	39.678	
	**Negative results**								
chr20	3896885	3898990	hsa-mir-103	709.63	864.88	-155.25	1.008	-15.525	0.824
chr7	1.31E+08	1.31E+08	hsa-mir-29a	725.13	853.00	-127.88	3.882	-12.788	
chr7	1.31E+08	1.31E+08	hsa-mir-29b	725.13	853.00	-127.88	2.628	-12.788	
chr16	56891111	56894637	hsa-mir-138	762.25	794.50	-32.25	2.793	-3.225	
chr12	7070317	7078019	hsa-mir-141	257.48	233.22	24.26	0.922	2.426	
chr12	7070317	7078019	hsa-mir-200c	257.48	233.22	24.26	1.062	2.426	
chr3	49053208	49062679	hsa-mir-191	207.78	180.64	27.14	1.662	2.714	
chr3	49053208	49062679	hsa-mir-425	207.78	180.64	27.14	1.500	2.714	
chr22	46507241	46509665	hsa-let-7b	862.27	830.07	32.20	1.025	3.220	
chr1	1098001	1107489	hsa-mir-200a	513.71	469.62	44.10	1.067	4.410	
chr1	1098001	1107489	hsa-mir-200b	513.71	469.62	44.10	1.028	4.410	
	**Control**								
chr3	1.68E+08	1.68E+08	hsa-mir-551b	91.67	860.33	-768.67	-0.08867	-76.867	-0.111
chr13	50618255	50628337	hsa-mir-15a	689.50	864.50	-175.00	0.03667	-17.500	
chr7	1.29E+08	1.29E+08	hsa-mir-183	712.59	766.88	-54.29	0.2	-5.429	
chr7	1.29E+08	1.29E+08	hsa-mir-96	712.59	766.88	-54.29	-0.195	-5.429	
chr17	28439097	28449190	hsa-mir-423	201.42	194.16	7.26	-0.31	0.726	
chr17	57223497	57233582	hsa-mir-301a	222.05	179.60	42.45	-0.485	4.245	
chr7	99686391	99696470	hsa-mir-93	557.63	496.57	61.07	-0.565	6.107	
chr3	52297294	52307377	hsa-let-7g	788.40	724.60	63.80	0.255	6.380	
chr7	99686616	99696697	hsa-mir-106b	705.13	628.96	76.17	-0.05667	7.617	
chr16	14392824	14402906	hsa-mir-193b	640.40	560.27	80.13	0.44	8.013	
chr11	1.11E+08	1.11E+08	hsa-mir-34b	770.54	387.17	383.38	-0.302	38.338	
